# A Metal–Organic Framework Nanosheet‐Assembled Frame Film with High Permeability and Stability

**DOI:** 10.1002/advs.201903180

**Published:** 2020-02-25

**Authors:** Chuanhui Huang, Cong Liu, Xiangyu Chen, Zhenjie Xue, Keyan Liu, Xuezhi Qiao, Xiao Li, Zhili Lu, Lan Zhang, Zhenyu Lin, Tie Wang

**Affiliations:** ^1^ Ministry of Education Key Laboratory for Analytical Science of Food Safety and Biology Fuzhou University Fujian 350116 China; ^2^ Beijing National Laboratory for Molecular Sciences Key Laboratory of Analytical Chemistry for Living Biosystems Institute of Chemistry Chinese Academy of Sciences(CAS) Beijing 100190 China; ^3^ University of Chinese Academy of Sciences Beijing 100049 China; ^4^ Key Laboratory of Materials Processing and Mold Ministry of Education Zhengzhou University Zhengzhou 450002 China

**Keywords:** composite catalysts, heteroepitaxial growth strategies, metal–organic frameworks, nanosheets, nanosheet‐assembled frame film, thin films

## Abstract

The engineering of metal–organic frameworks (MOFs) into membranes and films is being investigated, to transform laboratory‐synthesized MOFs into industrially viable products for a range of attractive applications. However, rational design and construction of highly permeable MOF thin films, without trade‐offs in terms of structural mechanical stability, remains a significant challenge. Herein, a simple, general strategy is reported to prepare thin MOF nanosheet (NS)‐assembled frame film via heteroepitaxial growth from metal hydroxide film. As the thin MOF NS‐assembled film significantly enhances the permeability of mass though the film, the resultant gold nanoparticle (Au NP)@MOF film exhibits much higher catalytic efficiency than the Au NP@MOF bulk film. Meanwhile, the unique framework of the MOF NS‐assembled film resists torsion and collapse, so the composite catalyst exhibits long‐term stability.

## Graphical abstract

A thin metal–organic framework nanosheet‐assembled frame (MOF NAF) film is prepared via a facile heteroepitaxial growth strategy. The thin MOF NAF film significantly enhances the permeability of the film and the unique framework structure endows the MOF NAF film with high mechanical stability.

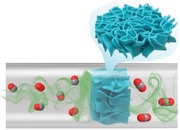

Metal–organic frameworks (MOFs),^[^ 1 ^]^ which have a high surface area and uniform cavities, as well as structural and functional tunability, have been widely used for various applications, including catalysis,^[^ 2, 3 ^]^ sensing,^[^ 4, 5 ^]^ storage and separation,^[^ 6, 7 ^]^ devices,^[^ 8 ^]^ drug delivery and imaging.^[^ 9 ^]^ As one of the most valuable forms for practical use, MOF‐based films have attracted more and more interest because they could serve as highly versatile, low‐energy alternative materials for chemical sensors, separators, and catalysts.^[^ 10, 11, 12 ^]^ In MOF film‐based applications, the selection of MOFs with pores of appropriate size, shape, and chemistry could allow for adsorption and separation with high selectivity. However, the thick MOF films still suffer from low flux due to the low diffusion rates of molecules in the long and narrow channels of MOFs (**Figure** **1**a,b).^[^ 13, 14 ^]^

**Figure 1:**
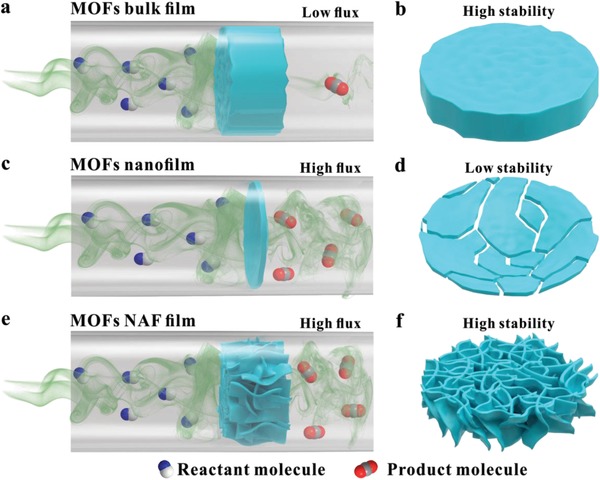
a,b) Schematic overview of the permeability and mechanical stability of the MOF bulk film under a catalyzed gas phase reaction. c,d) Schematic overview of the permeability and mechanical stability of the MOF nanofilm under a catalyzed gas phase reaction. e,f) Schematic overview of the permeability and mechanical stability of the MOF NAF film under a catalyzed gas phase reaction.

Pioneering efforts have demonstrated that preparing ultrathin MOF nanosheet (NS)‐based film is an effective strategy for maximizing the permeance of MOF films.^[^ 12 ^]^ For example, ultrathin Zn_2_(bim)_4_ NS‐ and MAMS‐1 NS‐based nm‐thick molecular sieving membranes showed high gas permeation flux.^[^ 15, 16 ^]^ According to Fick's Law, the Knudsen diffusion flow passing through the micropores of MOFs follows the rule:^[^ 17 ^]^ *J* ∝ 1/*L, where J—permeability; L—film thickness*. Therefore, the thinner the prepared film, the higher its permeability (Figure 1c). The construction of MOF NSs in very thin layers benefits the permeance of molecules, but restricts mechanical stability and cyclic utilization due to the very low elastic modulus of the MOF NSs. The soft nanosheets are always suffering from severe structure deterioration or fragmentation during exfoliation, application, and recollection. This greatly limits their applications in some cases (Figure 1d).^[^ 18, 19 ^]^ Our previous study revealed that while the NSs are assembled into an intersecting structure, the stress or strain can be effectively distributed, thus the NSs‐based structure obtaining high mechanical stability.^[^ 20 ^]^ This stimulates us to synthesize thin MOF NSs‐based films with high permeance and assemble the MOF NSs into frame film structure with high mechanical stability.

Numerous strategies for the fabrication of continuously MOFs films, composed of an uninterrupted, pure layer have been developed.^[^ 21 ^]^ One of the most economical and practical strategies for the large‐scale fabrication of MOFs film is the direct conversion of the ceramic precursors films into MOFs films at room temperature.^[^ 22, 23, 24, 25 ^]^ Herein, we design and construct a CuBDC nanosheet‐assembled frame (NAF) film using a heteroepitaxial growth method and set it as catalyst. The effective thickness for passage of molecules through the NAF film is reduced to the nanometer level (Figure 1e). Impressively, by encapsulating Au NPs into thin CuBDC NAF film, the Au NP@NAF composites exhibit much higher catalytic properties than Au NPs encapsulated within CuBDC bulk film. Meanwhile, the CuBDC NSs are interwoven with each other to realize an assembled frame that is conducive to dispersing stress and strain. The NAF exhibits much higher mechanical stability than freestanding NSs and shows excellent recyclability in catalytic reactions (Figure 1f). Our recyclability experiments revealed that the Au NP@NAF catalyst could maintain its morphology and structure during reactions. This versatile, general strategy could be used as a blueprint for controlled and economical synthesis of metal nanoparticle @NAF film.

With due consideration of the designed geometric structure described above, the CuBDC NAF film was synthesized through an accessible two‐step approach. First, copper hydroxide nanostrands films were synthesized in the previously reported manner, with minor modifications (Figure S1, Supporting Information).^[^ 26 ^]^ The copper hydroxide nanostrand thin film was subsequently transformed into a NAF film via reaction with organic ligands at low temperatures and low water fractions (**Figure** **2**a,c and Figures S2–S4, Supporting Information). Scanning electron microscopy (SEM) images show that the copper hydroxide film was converted into NAF film with a thickness of ≈3.0 µm; the NAF film consists of interlaced single‐crystal NSs with thickness ≈25 nm (Figure 2e). Furthermore, due to a change in the reaction conditions, the bulk film with a thickness of ≈1.2 µm, and a transitional CuBDC film (B‐NAF) with a thickness of ≈2.5 µm, were manufactured for comparison (Figure 2b–d; Figures S5–S7, Supporting Information). The ultraviolet–visible diffuse reflectance spectra indicate an apparent decrease in light absorbance from bulk to NAF film, confirming that the film became thinner from bulk to NAF (Figure S5c, Supporting Information). It is worth noting that the NAF film features a Brunauer–Emmett–Teller (BET) surface area (*S*_BET_) of 67.13 m^2^ g^−1^, which is over three times higher than that of the bulk film (*S*_BET_ = 19.73 m^2^ g^−1^), and higher than the well‐dispersed CuBDC NSs prepared via the bottom‐up method (Figure S5d, Supporting Information).^[^ 27 ^]^

**Figure 2:**
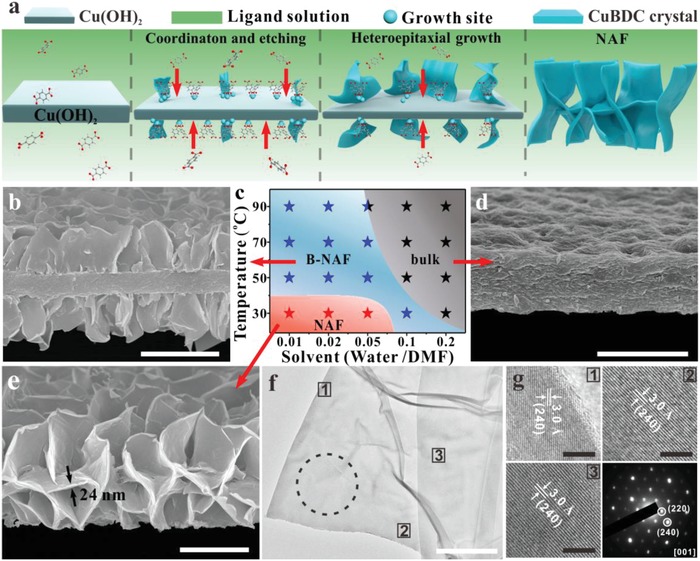
a) Schematic view of the mechanism for the preparation of a CuBDC NAF film. b) Cross‐sectional SEM image of the CuBDC B‐NAF product. c) Phase diagram that correlates the solvent composition (horizontal ordinate; the volume of DMF is 10 mL) and reaction temperature (vertical ordinate). d) Cross‐sectional SEM image of the CuBDC bulk product. e) Cross‐sectional SEM image of the CuBDC NAF product. f) TEM images of the CuBDC NS from the NAF product. g) HRTEM images of the white square shown in f) and the SAED pattern (black circle). The diameter and pore sizes of the nylon 66 membrane are 47.0 mm and 0.22 µm, respectively. Scale bars are 2 µm for (b,d,e), 1 µm for (f), and 2 nm for (g).

The effects of the dissolution and coordination rates on the products can be determined from the phase diagram, which summarizes the products generated under different solvent compositions and reaction temperatures (Figure 2c; Figure S8, Supporting Information). The NAF can only be found at low temperatures and low water fractions, while higher reaction temperatures and water fractions result in the formation of bulk materials. High temperature and water fractions are believed to increase the dissolution and coordination rate, leading to the formation of bulk film, while low dissolution and coordination rates lead to the formation of NAF film (Figure S9, Supporting Information). Furthermore, the NAF appears to have a low concentration of ligands during the reaction, whereas the bulk film has a high concentration (Figure S10, Supporting Information). This suggests that the low coordination rate between the copper ions and organic ligands leads to the formation of NAF, and that this is higher for the bulk sample.

On the basis of the above‐described experimental results, the growth of NAF films is precisely controlled by a heteroepitaxial growth mechanism,^[^ 28 ^]^ in which metal hydroxide can be converted into MOF crystals accompanied by strongly localized recrystallization (Figure 2a; Note S1, Supporting Information). Previous work revealed that the heteroepitaxial growth mechanism would restrict the orientation of growth between the MOFs and copper hydroxide nanostrands surfaces, that is, the *a* and *b* axis of MOF crystals should align with the *c* and *a* axis of a copper hydroxide substrate, respectively (See Figure S11, Supporting Information, for details).^[^ 24, 25 ^]^ Moreover, the interplanar spacing (3.0 Å for (240) planes) and diffraction spots from (240) planes in the electron diffraction pattern confirm the heteroepitaxial growth of CuBDC NAF on copper hydroxide, which is in good agreement with previously reported results (Figure 2f,g).^[^ 24 ^]^ To further validate the above mechanism for the construction of NAF, some other MOFs either with the Cu^2+^ or the H_2_BDC ligand are synthesized via the same strategy (Figure S12, Supporting Information). As expected, the NAF morphologies would only occur while the maximum lattice mismatch between the MOF crystal and the inorganic substrate is less than 1.8%. For bulk film, the growth process followed the conventional “dissolution‐precipitation mechanism,^[29,30]^ which has been observed in other sacrificial‐template synthetic strategies of MOF nanostructures (Figure S13a and Note S2, Supporting Information). The B‐NAF synthesis process shares the same growth mechanism as NAF during the early stages of the reaction. However, halfway through the reaction, the closely packed NSs cover the surface of the copper hydroxide and promote further localized conversion into the bulk shape (Figures S13b and S14, Supporting Information). The NAF film fabrication process does not any release metal ions into the solution while for the fabrication of bulk film, a large number of Cu^2+^ cations in the solution makes it easy for CuBDC nanocrystals to form therein (Figures S13c and S15, Supporting Information). The low residual cations in solution also demonstrated that the ceramic‐to‐MOF conversion efficiency is as high as 99.7%. Furthermore, the pH values were found to decrease gradually with the concentration of Cu^2+^ cations, which can be attributed to an in situ acid–base interaction between the copper hydroxide and H2BDC (Figure S13d, Supporting Information).

Thinning MOF bulk film into NAF film leads to changes in its mechanical properties, which must be taken into account. Many previous studies have found that the deterioration of the structure and morphological fragmentation of MOF NSs during synthesis and application is due to the relatively low Young's modulus of MOF NSs.^[^ 18, 31, 32 ^]^ To explore the mechanical properties, we deposited freestanding CuBDC bulk film and NAF film onto a Si wafer via a facile transfer process. The CuBDC NS thin films were prepared using the Langmuir–Schäfer method (Figures S16 and S17, Supporting Information).^[^ 33, 34 ^]^ As shown in one cycle of measurement of the mechanical properties, the indentation depth for NAF film (221.68 ± 19.13 nm) was only slightly increased from that of the bulk film (192.67 ± 32.18 nm) under the same load (500 uN), while the NS film exhibits a much greater indentation depth (376.26 ± 64.03 nm), even under lower load levels (200 µN) (**Figure** **3**a). After obtaining seven measurements at various sites on the NAF film, the average Young's modulus and hardness were 11.91 ± 1.24 and 0.36 ± 0.07 GPa, respectively (Figure 3b; Table S1, Supporting Information). The mechanical property data are nearly six times higher than those of the NS film (2.09 ± 0.35 and 0.06 ± 0.02 GPa, respectively). These exceptionally high Young's moduli are well above those of various bulk MOF crystals (Figure S18 and Table S2, Supporting Information). The stress simulation revealed the same results. Under the same external stress, the simulated displacements of bulk film (400 nm) and NS film (25 nm) are 0.23 nm and 16.17 nm, respectively (Figure 3c,d). Surprisingly, while the NSs assembled into scaffolds (NAF film), the simulated displacement was only 1.53 nm, which was 90.5% less than that of the NS film (Figure 3e). Significant breakage occurs in the freestanding nanofilm, while the bulk and NAF films retain their integrity under the same external stress. Such porous NAF films with high Young's moduli and hardness, constructed via ultrathin NS units, are excellent candidate materials for improving mass transfer and maintaining the stability of the structure in catalytic applications.

**Figure 3:**
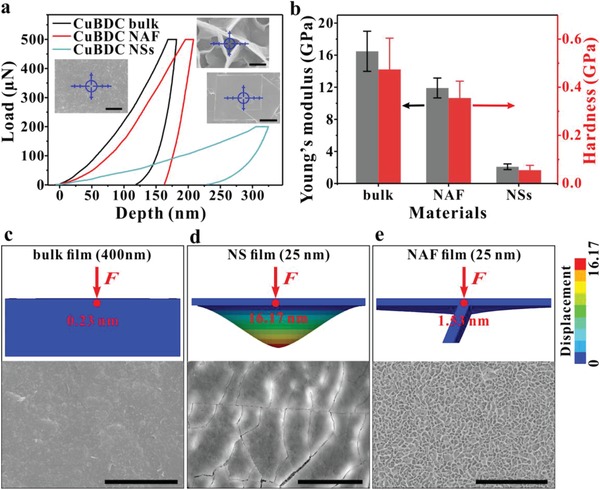
a) Typical load–displacement nanoindentation curves for different CuBDC film structures. The inset SEM images show the surfaces of the CuBDC bulk, NAF and NS for the test film samples on Si/SiO_2_ substrates; the blue crosshair represents the test site. b) Young's modulus and hardness for different CuBDC film structures. Error bars represent the standard deviation of three replicate samples. c–e) Model of the CuBDC bulk film, CuBDC NS film, and NAF film and their simulated cloud pictures of the displacement distribution profiles of film cross‐sections under external stress *F* (top). Surface SEM image of CuBDC bulk film, CuBDC NS film and CuBDC NAF film after loading an external 800 µN force (bottom). Scale bars are 200 nm for the inset in (a), and 20 µm for (c)–(e).

The structure of CuBDC NAF film is believed to significantly enhance molecule permeation flux. The CuBDC bulk and CuBDC NAF are modeled as a single thick plate (800 nm thick) and thin NS (25 nm thick)‐assembled frameworks, respectively. Under steady state conditions, the entire hydrodynamic flow profile can be obtained using computational fluid dynamics (CFD) analysis and the simulated maximum outlet velocities of the eight pores in the cross‐section of the two models are compared with each other. The maximum outlet velocity for the NAF is 13.1 to 31.0 times higher than that of the bulk, indicating that the structure of the NAF is superior at facilitating mass diffusion through MOF crystals (**Figure** **4**a,b,d). It is well known that the pressure loss along the path (Δ*P*_λ_) in the rigid holes of MOFs can be defined by the following equation:^[^ 35 ^]^ Δ*P_λ_* = λ*l/d*ρv ^2^/2, where λ—resistance coefficient; *l*—length; *d*—diameter; ρ—density of liquid; and *v*—flow velocity. As the pressure loss along the path is positively correlated with the length of pipe (*l)*, *ΔP_λ_* is obviously higher through the pores of bulk than through NAF, while the initial flow velocity is the same. Moreover, the fluid flow through a pipe is defined by the Bernoulli equation:^[^ 36 ^]^ *C* = *P* + *ρv^2^*/2 + *ρgh*, where *C*—constant; *P*—potential energy of pressure; *ρv^2^*/2—kinetic energy; and *ρgh*—potential energy of gravity. As the inlet and outlet of the pipe have the same height and external pressure, the potential energy of gravity and pressure remain the same, so all of the pressure loss along the path is converted into a loss of kinetic energy. It can readily be seen that the decrement in outlet velocity of the NAF is lower than that of the bulk. The remarkable thinning of the film from bulk to NAF leads to a precipitous decrease in pressure loss along the path through the pores, which is responsible for the improved mass transfer performance. The hydrodynamic flow profile of the NS film was also characterized, and the simulated maximum outlet velocity of the pores was found to be very similar to that of the NAF. This demonstrates that the high permeability is mainly derived from the ultrathin effective thickness of the NAF film (Figure 4c,d). As shown in the permeability test (Figure 4e), the gas flux of thin NAF membrane is obviously superior to bulk membrane which is in good agreement with theoretical simulation. The CO_2_ fluxes of CuBDC NAF film are enhanced 3.8‐fold, 3‐fold, and 2.6‐fold compared with CuBDC bulk film at operating pressure of 1, 2, and 3 bar, respectively.

**Figure 4:**
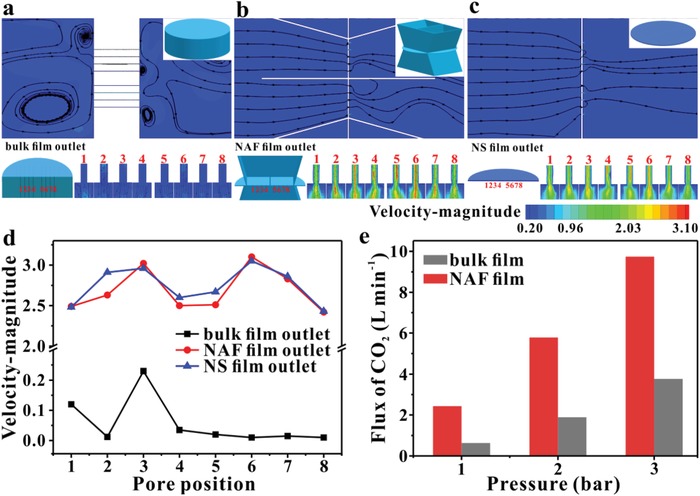
a–c) Velocity magnitude and flow pattern gained from computational fluid dynamics (CFD) analysis of the bulk, NAF and NS model and the velocity profile inside the outlet of the pores. Model of 800 nm thick bulk film, NAF model and NS model (inset). d) The statistical maximum outlet flow velocities from the eight pores in (a–c). e) Comparison of the CO_2_ flux value of different films at different operating pressure.

As a proof of concept, gold nanoparticles (Au NPs) were encapsulated into CuBDC NAF film for CO oxidation, to verify that the NAF substrate indeed facilitates fast mass diffusion, thus promoting heterogeneous catalysis. Well‐defined and polycrystalline Au NPs, 13 ± 1 nm in size, were synthesized as reported previously using exposed (111) facets (**Figure** **5**a; Figure S19, Supporting Information).^[^ 37 ^]^ By mixing Au NPs into the copper hydroxide nanostrands film beforehand, we obtained three different morphologies of Au NP@CuBDC films (Figure 5b,c; Figure S20, Supporting Information). The thickness of the NSs in Au NP@NAFs films is ≈25 nm; this was not affected by the amount of Au NPs (Figure S21, Supporting Information). Only very few of Au atoms are detected by XPS spectra, revealing the Au NPs are well encapsulated within the NSs in the Au NP@NAFs films (Figure S22, Supporting Information). This confirms that this synthesis strategy is suitable for the fabrication of MNP@NAFs composite films. The BET surface area of Au NP@NAFs is as high as 57.0 m^2^ g^−1^, suggesting the existence of Au NPs do not affect the porosity of NAF (Figure S23, Supporting Information).

**Figure 5:**
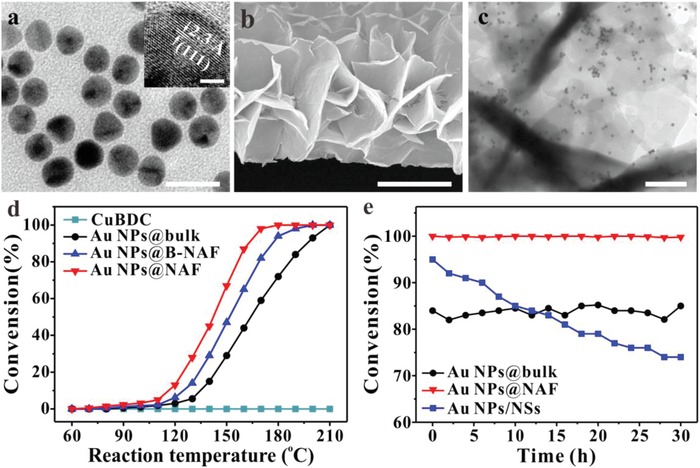
a) The TEM and high resolution TEM (HRTEM, inset) images of gold nanoparticles (Au NPs). b) Cross‐sectional SEM image of the Au NP@NAF composites film. c) TEM image of the Au NP@NAF NS. d) Conversion versus temperature for CO oxidation catalyzed by Au NP@CuBDC composites. e) Conversion at 190 °C against time. The data presented here are the average values recorded from three individual samples, with an average error of approximately 5%. Scale bars are 25 nm for (a), 2 nm for the inset, 2 µm for (b), and 500 nm for (c).

Upon heating the nanocomposite in diluted reaction gas containing a mixture of carbon monoxide and oxygen, the Au NP@NAF film had the highest catalytic efficiency among the three catalysts, as expected (Figure 5d). In the case of the Au NP@NAF composite film, the temperature of half conversion (*T*_50%_) was 143 °C and that of full conversion was 180 °C, while the Au NPs@B‐NAF and Au NP@bulk showed 50% CO conversion at 151 and 164 °C, and full conversion at 200 and 210 °C, respectively. Compared with Au NP@bulk film, the higher catalytic efficiency of Au NP@NAF film can be attributed to the fast mass diffusion through the film. Furthermore, the uniquely assembled structure endows Au NP@NAF with excellent mechanical stability, with no obvious decrease in yield during a catalytic reaction at 190 °C for up to 30 h (Figure 5e). The overall morphology indicates that the NAF NSs maintain their assembled frame, and the CuBDC‐covered Au NPs were well dispersed in the MOF NSs (Figures S24 and S25, Supporting Information). The nitrogen adsorption and desorption isotherms and X‐ray diffraction (XRD) pattern show that the used catalyst preserved the crystallinity and porosity of the as‐synthesized catalyst well, suggesting long‐term stability of the catalyst (Figures S23 and S26, Supporting Information). The Au NP@bulk also exhibits high stability, even if its catalytic efficiency is lower than Au NP@NAF (Figure S27, Supporting Information). For comparison, a contrast sample is prepared by simply mixing Au NPs and the CuBDC NSs (referred to as Au NPs/CuBDC NSs). Although the Au NPs/CuBDC NSs composites present high catalytic efficiency at the beginning, the catalyst suffer from severe breakage and agglomeration with a significant drop in catalytic efficiency during a catalytic reaction (Figure 5e; Figure S28, Supporting Information).

In summary, we developed a facile strategy for constructing NAF film using a heteroepitaxial growth method and simultaneously encapsulating Au NPs to be functionalized in Au NP@MOF composite film. The NAF film maximizes the mass transfer rate through the narrow MOF pores, and the Au NP@NAF film exhibits much higher catalytic efficiency than the Au NP @bulk film. Our CFD simulations confirmed that the thinner film could increase the permeability of the MOF pores. Moreover, the NAF film exhibits much higher mechanical stability than the NS film, which is conducive to recycling of the catalyst. Our study opens up a new avenue for developing powerful and mechanically stable multifunctional MOF‐based films, and demonstrates their potential applicability to industrial catalysis.

## Experimental Section

##### Preparation of CuBDC Bulk, B‐NAF, and NAF

Copper hydroxide precursor were synthesized following a synthesis strategy described by Peng et al., with minor modifications.^[^ 26 ^]^ Equal volumes of 4 mm copper nitrate solution and 1.4 mm aminoethanol solution were rapidly mixed and aged at 60 °C for 1 h. Filtering 60 mL of the mixture solution through a nylon 66 microporous membrane left a light‐blue thin film on the membrane. To prepare the three different morphologies of MOF, the obtained film was reacted with 10 mL of a 10 g L^−1^, 10 mL of a 1.0 g L^−1^, and 50 mL of a 0.2 g L^−1^ terephthalic acid solution (DMF/water: 10:0.1 = v/v) at room temperature for 6 h to prepare CuBDC bulk, B‐NAF, and NAF, respectively. A pure MOF thin film typically formed after 6 h at room temperature. The membrane holding the pure MOF thin film was vacuum‐dried at 120 °C overnight to remove residual solvent.

##### Preparation of CuBDC NSs

CuBDC NSs were synthesized following a three‐layer synthesis strategy described by Gascon et al.^[^ 27 ^]^ Typically, in a 20‐mL glass test tube, an organic ligands solution composed of 30 mg of H_2_BDC dissolved in a mixture of 2 mL of DMF and 1 mL of CH_3_CN was poured into the bottom of the tube. Over this solution, a mixture of 1 mL of DMF and 1 mL of CH_3_CN was carefully added to prevent premature mixing of the two solutions containing the precursors. Finally, a metal precursor solution composed of 30 mg of Cu(NO_3_)_2_·3H_2_O dissolved in a mixture of 1 mL of DMF and 2 mL of CH_3_CN was also carefully added to the tube as the top layer. The synthesis proceeded at 40 °C for 24 h in static conditions, and the resulting precipitate was collected by centrifugation and washed consecutively three times with DMF (5 mL each step) followed by another three times with CHCl_3_ (5 mL each step).

##### Mechanical Property Measurement

For comparison, the CuBDC NSs were synthesized as reported previously. Detailed methods for the synthesis of CuBDC NSs is presented in the Supporting Information. The mechanical properties were measured using a nanoindenter (TriboScope; Hysitron, Minneapolis, MN, USA) with a Berkowich diamond tip, which is a three‐sided pyramid with a tip radius of ≈150 nm. The indentation depth was set in the film thickness range of 10–20% to minimize the influence of the substrate on the samples. Seven repeated indentations were made for each sample. A typical load‐displacement curve was obtained at a constant loading/unloading rate of 100 µN s^−1^.

##### Permeability Test

The permeability test measured by a fully automatic bubble pressure method filter membrane pore size analyzer (3H‐2000 PB, BeiShiDe Instrument Technology Co., Ltd., China). Two CuBDC coated nylon 66 membrane with a diameter of 20 cm are used for test. The operating temperature was 25 °C and operating pressure was 1, 2, and 3 bar.

##### Preparation of Au NPs (≈13 nm)

First, Au NPs with an average diameter of 13 nm were prepared using the citrate reduction method.^[^ 38 ^]^ Briefly, a solution of sodium citrate (10 mL; 38 mm) was added to a rapidly stirred boiling aqueous solution of HAuCl_4_ (100 mL; 1 mm). After an additional 15 min of boiling, the solution was allowed to cool to room temperature, filtered and stored in the refrigerator (4 °C). The concentration of 13 nm Au NPs was calculated to be approximately 0.3 mg mL^−1^.

##### Preparation of Au NP @CuBDC Composite Thin Film

A 1.2‐mL amount of the prepared 13‐nm Au NPs colloids was mixed with 45 mL of copper hydroxide nanostrand solution and ultrasonicated for approximately 10 min. After filtering the mixture solution on a nylon 66 microporous membrane, an Au NP/copper hydroxide thin film was obtained. The obtained film was reacted with 10 mL of a 10 g L^−1^, 10 mL of a 1.0 g L^−1^, and 50 mL of a 0.2 g L^−1^ terephthalic acid solution (DMF/water: 10:0.1 = v/v) at room temperature for 6 h to prepare Au NP@bulk, Au NP@B‐NAF, and Au NP@NAF, respectively. The membrane holding the Au NP@CuBDC composite thin film was vacuum‐dried at 120 °C overnight to remove the residual solvent.

##### Preparation of Au NPs/CuBDC NS Composite

First, the Au NPs solution was concentrated to 1.5 mg mL^−1^, then 1.0 mL Au NPs solution was added to CuBDC NSs ethanol solution (5.0 mL; 30 mg), and then the mixture was ultrasounded for 5 min. Finally, the Au NP@CuBDC composite was vacuum‐dried at 120 °C overnight to remove the residual solvent.

##### CO‐Catalyzed Oxidation by Au NP@CuBDC Composite Thin Film

First, 100 mg of Au NP (13 nm) @CuBDC composite thin films with approximately 5 mg of Au NPs were loaded into the middle of a quartz tube mounted on a tube furnace. A gas flow (200 mL min^−1^) mixture of CO (1%), O_2_ (7%), and N_2_ (92%) was used. The composition of the effluent gas was analyzed using gas chromatography. The temperature for 50% conversion (*T*_50%_) was selected as an index for evaluating the activity of the catalysts.

##### Simulation of Strain Profiles

The cloud picture of the displacement distribution profiles after processing was simulated using ANSYS engineering software (ANSYS, Inc., Canonsburg, PA, USA). For the NAF, the model used to simulate the cloud picture of the displacement distribution consisted of two plates made of 2D CuBDC NSs, of dimensions 1000 × 1000 × 10 nm^3^; the dihedral angle of the two plates converged at a perfect 60°. For the CuBDC bulk film, a model of dimensions 1000 nm × 1000 nm × 400 nm was used to simulate the cloud picture of the displacement distribution. The material properties were as follows: density, 1.637 × 10^−6^ ng nm^−3^;^[^ 39 ^]^ elasticity modulus, 5 GPa;^[^ 16 ^]^ and Poisson's ratio, 0.3.^[^ 40 ^]^ The plates were constrained as follows: the displacement of most external nodes of the constrained edges was zero in three directions. Simulated loads of 20 nN were applied to the plates at the center of 10 × 10 nm^2^ regions.^[^ 32 ^]^

##### Methodology for CFD Simulation

CFD simulations were carried out using the commercial software FLUENT (ANSYS Inc.). Ethyl acetate was used as a working fluid (a homogeneous, incompressible Newtonian fluid; ρ = 902.0 kg m^−3^, μ = 0.426 mPa s). Both models have pores at the same positions, and with the same shapes in the middle of the plate, suspended in a Newtonian liquid solution of the same viscosity. All molecular species in the solution are assumed to be able to diffuse (with a reduced diffusion constant) through the pores. All simulations were performed using an iterative, segregated solution method until a converged solution was obtained. A Navier–Stokes simulation was carried out using the FLUENT software.^[^ 41 ^]^ The Spalart–Allmaras turbulence model was used. To meet the parameter setting requirements of the software, all of the material sizes were expanded by a factor of 10^5^. The boundary conditions of the pressure inlet and pressure outlet were defined, and the adiabatic wall condition was adopted for the wall surface. In the calculation process, the mass flow at the orifice exit and the residual error were monitored. The calculation convergence criterion is as follows: the residual of the continuous equation, energy equation, momentum equation, and S‐A equation decreases by more than three orders of magnitude or the residual of the continuous iteration no longer decreases.

## Conflict of Interest

The authors declare no conflict of interest.

## Supporting information

Supporting Information
