# Supplementary material for: A Metal–Organic Framework Nanosheet‐Assembled Frame Film with High Permeability and Stability
Source: Adv Sci (Weinh). 2020 Feb 25;7(8):1903180. doi: 10.1002/advs.201903180 (PMC7175284; doi:10.1002/advs.201903180)
Supplement: Supplementary file 1 — Supporting Information [file ADVS-7-1903180-s001.pdf]

Copyright WILEY-VCH Verlag GmbH & Co. KGaA, 69469 Weinheim, Germany, 2020.

ADVANCED SCIENCE
Open Access

Supporting Information

for *Adv. Sci.,* DOI: 10.1002/advs.201903180

A Metal–Organic Framework Nanosheet-Assembled Frame Film with High Permeability and Stability

Chuanhui Huang, Cong Liu, Xiangyu Chen, Zhenjie Xue, Keyan Liu, Xuezhi Qiao, Xiao Li, Zhili Lu, Lan Zhang, Zhenyu Lin, and Tie Wang**

Copyright WILEY-VCH Verlag GmbH & Co. KGaA, 69469 Weinheim, Germany, 2020.

A metal-organic framework nanosheet-assembled frame film with high permeability and stability

Chuanhui Huang, Cong Liu, Xiangyu Chen, Zhenjie Xue, Keyan Liu, Xuezhi Qiao, Xiao Li, Lan Zhang, Zhenyu Lin, and Tie Wang**

WILEY-VCH

Materials and characterizations

Materials

All chemicals used were at least of analytical grade. Copper nitrate trihydrate ($Cu(NO_3)_2$•$3H_2O$), 2-aminoethanol (NH_2-CH_2CH_2OH), 1,3,5-benzenetricarboxylic acid (trimesic acid, H_3BTC), terephthalic acid (H_2BDC), 2,6-naphthalenedicarboxylic acid (H_2(2,6-NDC), 4,4′-biphenyldicarboxylic acid (H_2BPDC), 2-aminoterephthalic acid (NH_2-H_2BDC), nitroterephthalic acid (NO_2-H_2BDC) were purchased from Aladdin (Shanghai, China). The supports were Nylon 66 microporous membranes (Jin teng) with an average pore size of *ca* 220 nm and porosity of 50%. Ultrapure water (18.2 MΩ) produced by a Millipore direct-Q system (Millipore) was used throughout the experiments.

Characterizations

The products were characterized by XRD (model D/MAX2500; Rigaku, Tokyo, Japan) with Cu-Kα radiation at a scanning rate of 3° min^{-1}. The Fourier transform-infrared (FT-IR) spectra were measured using a Nicolet 6700 instrument (Thermo Fisher Scientific, Waltham, MA, USA). The morphologies were characterized by a S-4800 scanning electron microscope (Hitachi, Tokyo, Japan) equipped with energy-dispersive X-ray spectroscopy (EDX) analysis functionality. The transmission electron microscopy (TEM) images were obtained using H-800 (Hitachi) and JEM-2010 (JEOL, Tokyo, Japan) instruments at an accelerating voltage of 200 kV. The Au content was quantified by an Optima 7300 DV inductively coupled plasma-atomic emission spectrometer (ICP-AES; PerkinElmer, Waltham, MA, USA). The surface area and pore diameter were evaluated using a physisorption analyzer (model ASAP 2020M; Micromeritics, Norcross, GA, USA) at −196°C. Prior to the measurements, samples were degassed *in vacuo* at 180°C for at least 8 h. The S_{BET} was calculated using adsorption data at P/P_0 of 0.05–0.30. The pore size distributions (PSDs) were derived from the adsorption branches of the isotherms using the Barrett–Joyner–Halenda (BJH) model. The total pore volume (V_t) was estimated from the adsorbed amount at P/P_0 of 0.995. The TGA was carried out using a Pyris 1 TGA (PerkinElmer) with a nitrogen flow of 10 mL min^{-1}.

Methods

Preparation of Zinc hydroxide nanostrands film

Zinc hydroxide precursor were synthesized following a synthesis strategy described by Peng et al.[1] Equal volumes of 4 mM copper nitrate solution and 2.0 mM aminoethanol solution were rapidly mixed and aged at room temperature for 30 min. Filtering 60 mL of the mixture solution through a nylon 66 microporous membrane left a white thin film on the membrane.

Preparation of CuBDC bulk and NAF films on silicon wafer

First, the silicon wafers were sonicated for 15 min in acetone, ethanol and deionized water, respectively. The CuBDC film on Nylon 66 microporous membrane was heat at 100 ^oC for 15 min, then it was immersed in a cold acetone solution instantaneously. The CuBDC film was detached from the Nylon 66 microporous membranes spontaneously. Finally, the self-standing CuBDC film was deposited on the silicon wafer.

Preparation of CuBDC NS film on silicon wafer

First, the silicon wafers were sonicated for 15 min in acetone, ethanol and deionized water, respectively. The CuBDC NS films were deposited onto the aforementioned cleaned substrates through the Langmuir-Schäfer method.[2-5] Briefly, the synthesized CuBDC NSs were first dispersed in ethanol to obtain a colloidal suspension with a concentration of 1.0 mg mL^{-1}. Then the suspension was gently dropped onto the surface of water in a watch glass. After the CuBDC NSs spontaneously spread to form a thin film on water, the film was transferred onto a silicon wafer via the Langmuir-Schäfer method. Finally, the film-coated solid substrate was immersed into ethanol to remove the loosely deposited nanosheets prior to blowing it with N_2. The aforementioned procedure is defined as one deposition cycle. By repeating the aforementioned deposition procedure, MOF nanosheet films could be obtained.

Supplementary figures and tables

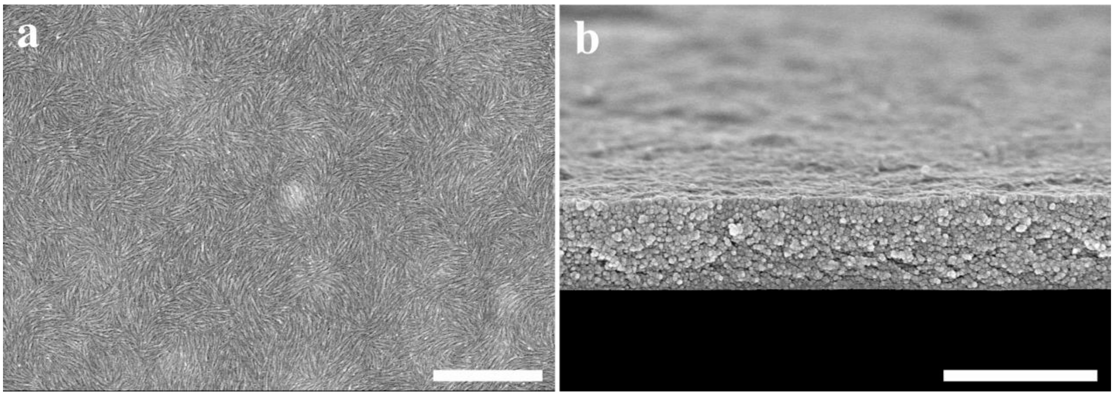

Figure S1. Surface and cross-section SEM images of the copper hydroxide nanostrands thin film precursors. Scare bars represent 1 μm for (a) and (b).

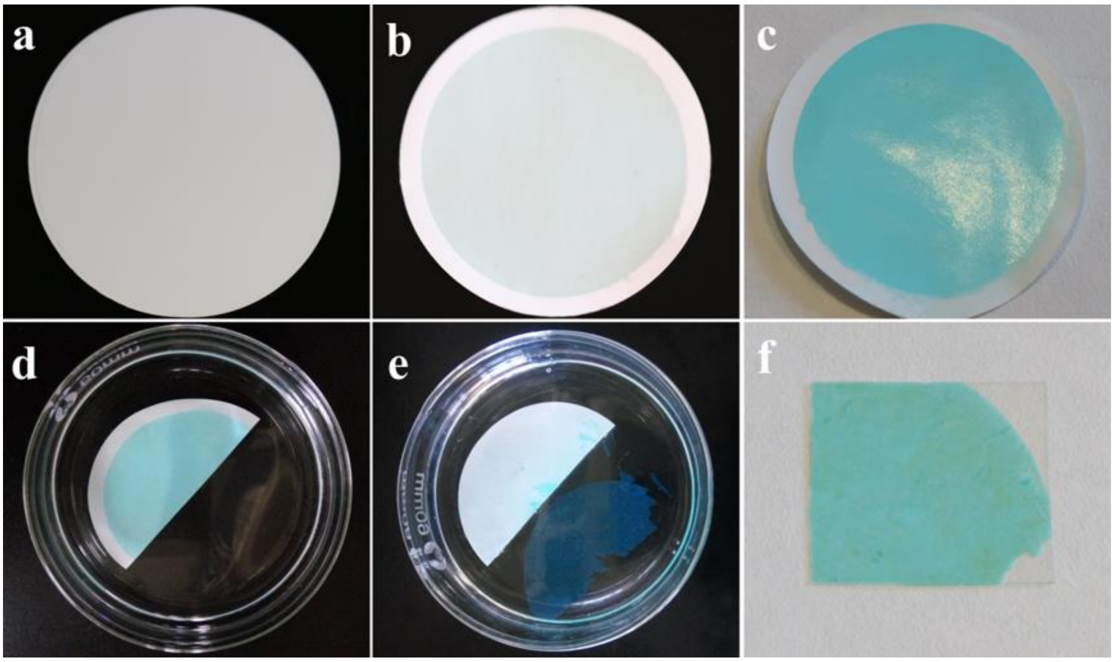

Figure S2. (a) Photographs of bare nylon 66 membrane, (b) copper-hydroxide- nanostrands coated nylon 66 membrane, (c) as-synthesized CuBDC NAF coated nylon 66 membrane, (d) the CuBDC NAF coated nylon 66 membrane dipped into cool acetone (the whole membrane was heated at 90 ^{o}C for 10 min before soak process), (e) the CuBDC NAF were detached from the nylon 66 membrane cool acetone, (f) The detached CuBDC NAF possess sufficient mechanical strength to be moved onto a coverslip. The diameter and pore size of nylon 66 membrane are 47.0 mm and 0.22 μm, respectively.

This result show that the CuBDC nanosheets are connected to each other to form a integrate membrane, and the CuBDC NAF could be easily deposited on any substrate which is highly desirable for practical applications.

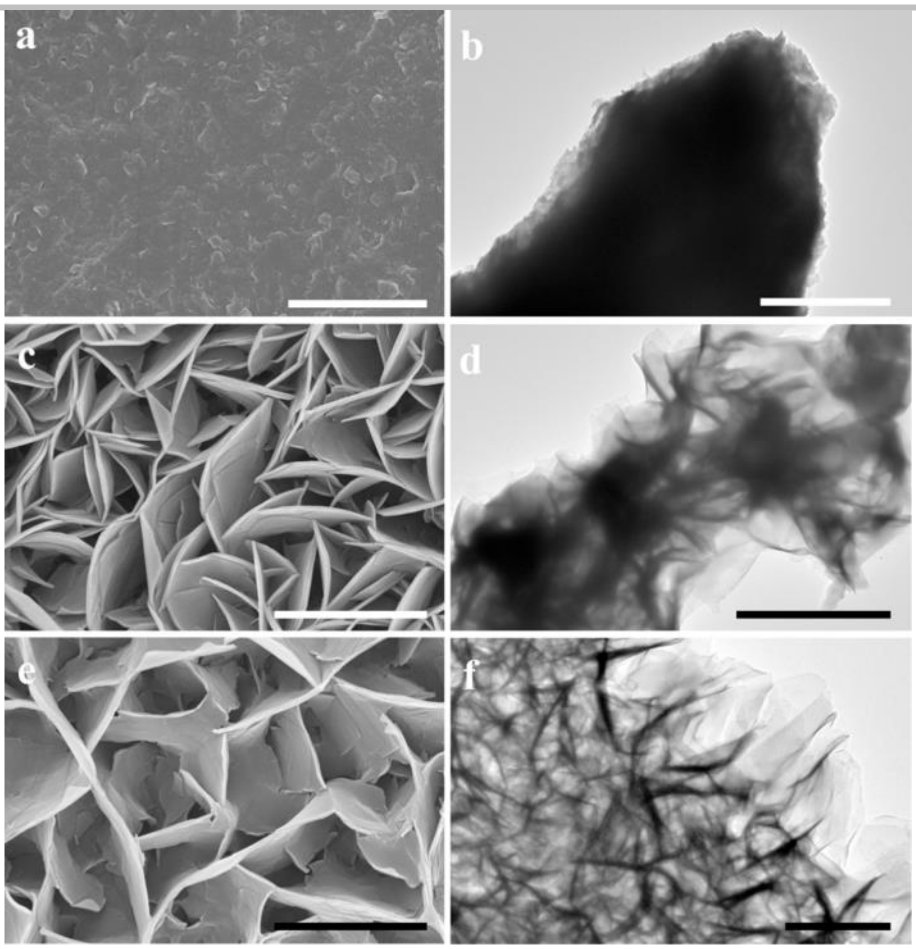

Figure S3. (a-b) Surface SEM and TEM images of the synthesized CuBDC bulk products, (c-d) Surface SEM and TEM images of the synthesized CuBDC B-NAF products. (e-f) Surface SEM and TEM images of the synthesized CuBDC NAF products. Scare bars represent 2 μm for (a-f).

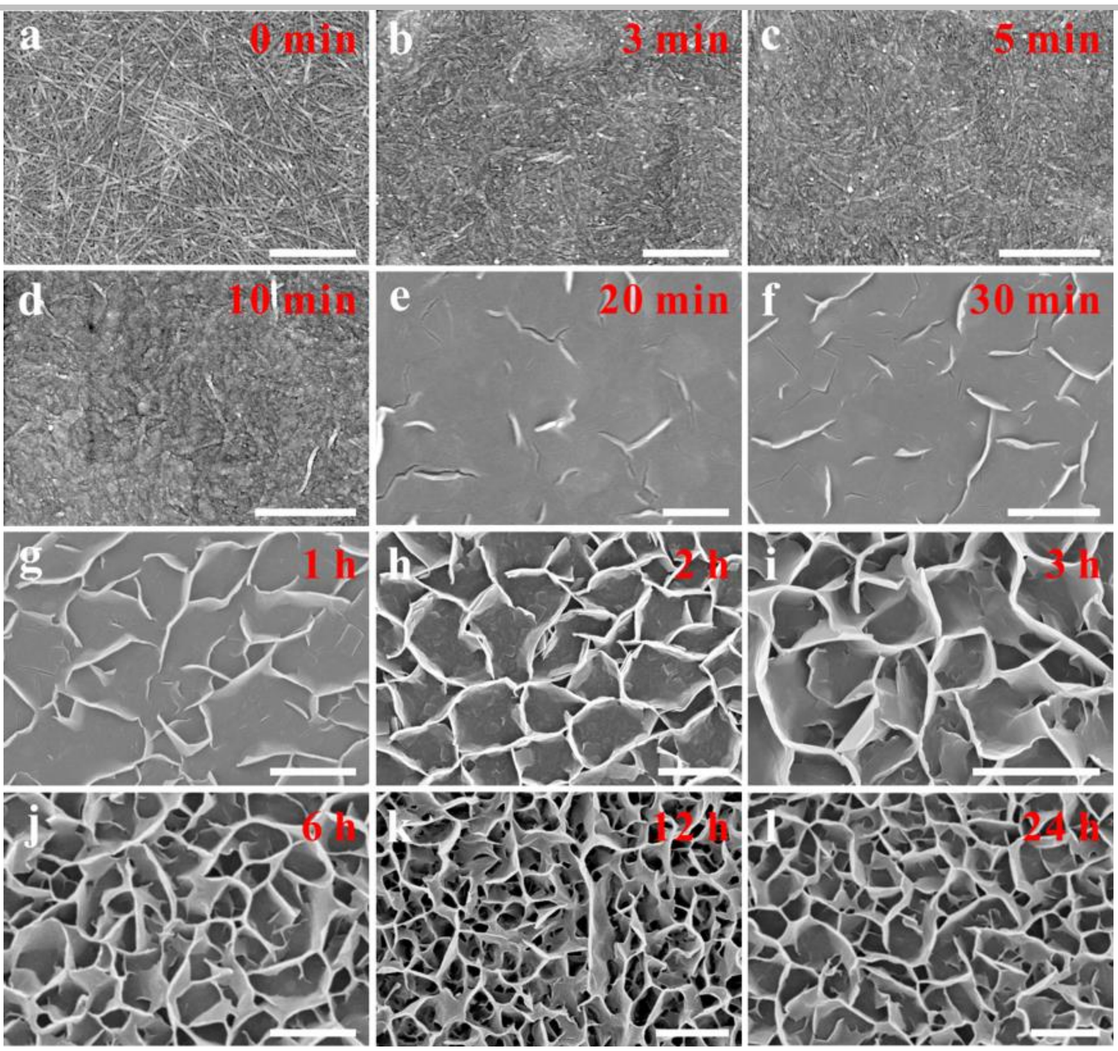

Figure S4. (a-l) Surface SEM images of copper hydroxide nanostrands thin film was transformed into CuBDC NAF at different reaction time (from 0 min to 24 h). Scare bars represent 500 nm for (a-e), 2 μm for (f-l).

In the initial time, the surface of copper hydroxide nanostrands thin film is quite flat and hundreds of copper hydroxide nanostrands can be see clearly. After the copper hydroxide nanostrands thin film was dipped into organic ligand solution, the morphology of the parent copper hydroxide nanostrands would readily evolve into blurry. Some small MOFs sprout emerged and stacked on the surface of film after 20 min of reaction at room temperature. When the process was prolonged to 2 h, it was observed that the nanosheets have covered the surface of the film and assembled into frame. The copper hydroxide nanostrands thin film has transformed into nanosheet assembled frame completely at 6 h.

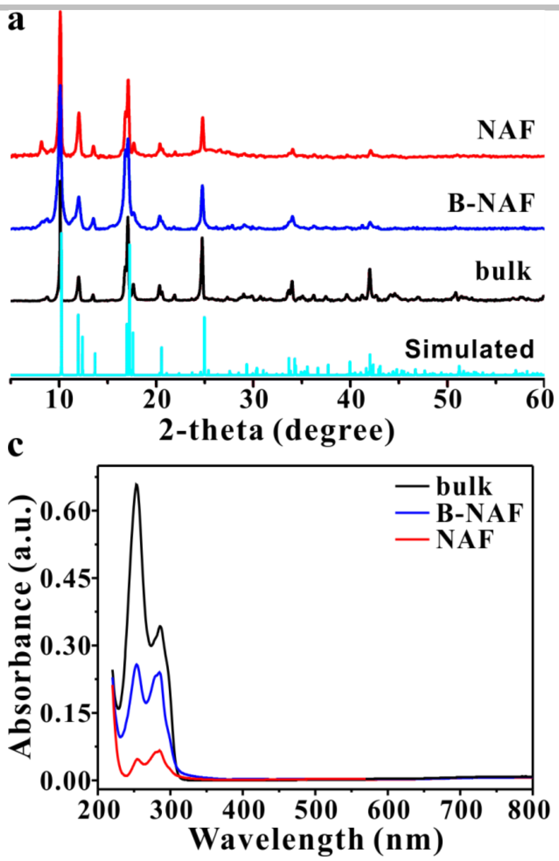

Figure S5. (a) XRD patterns of as-synthesized CuBDC bulk, B-NAF and NAF, (b) FT-IR spectra of as-synthesized CuBDC bulk, B-NAF and NAF, (c) UV-vis diffuse reflectance spectra of as-synthesized CuBDC bulk, B-NAF and NAF, (d) Nitrogen adsorption and desorption isotherms measured at 77 K of as-synthesized CuBDC bulk, B-NAF and NAF.

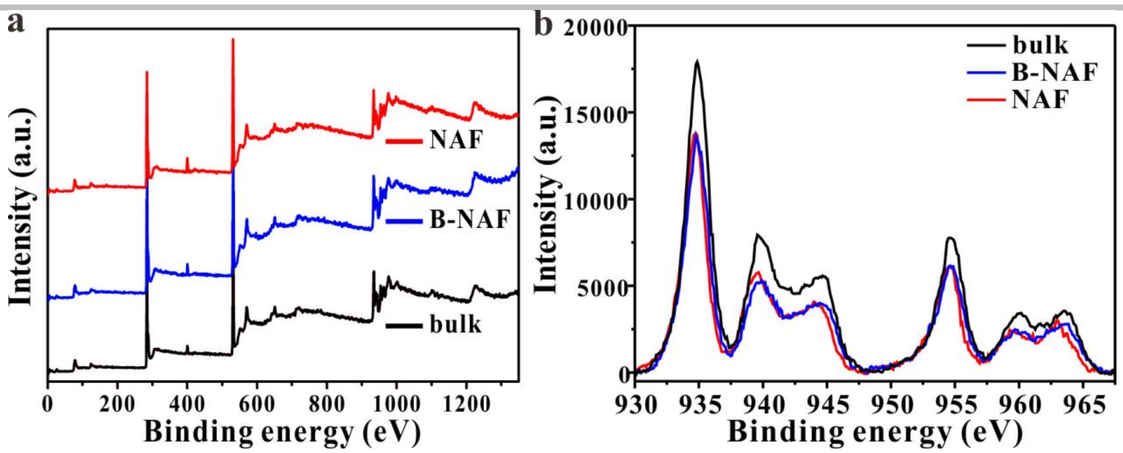

Figure S6. (a) XPS patterns of as-synthesized CuBDC bulk, B-NAF and NAF; (b) high-resolution XPS spectra of Cu 2p.

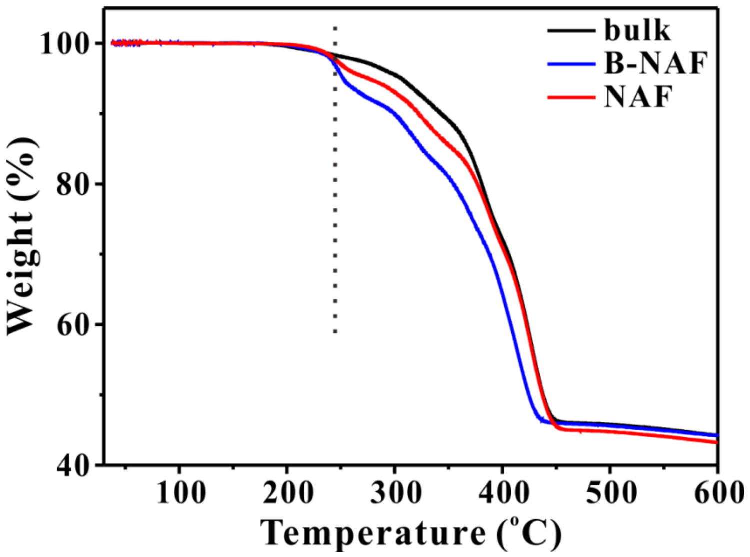

Figure S7. TGA curves of as-synthesized CuBDC bulk, B-NAF and NAF films.

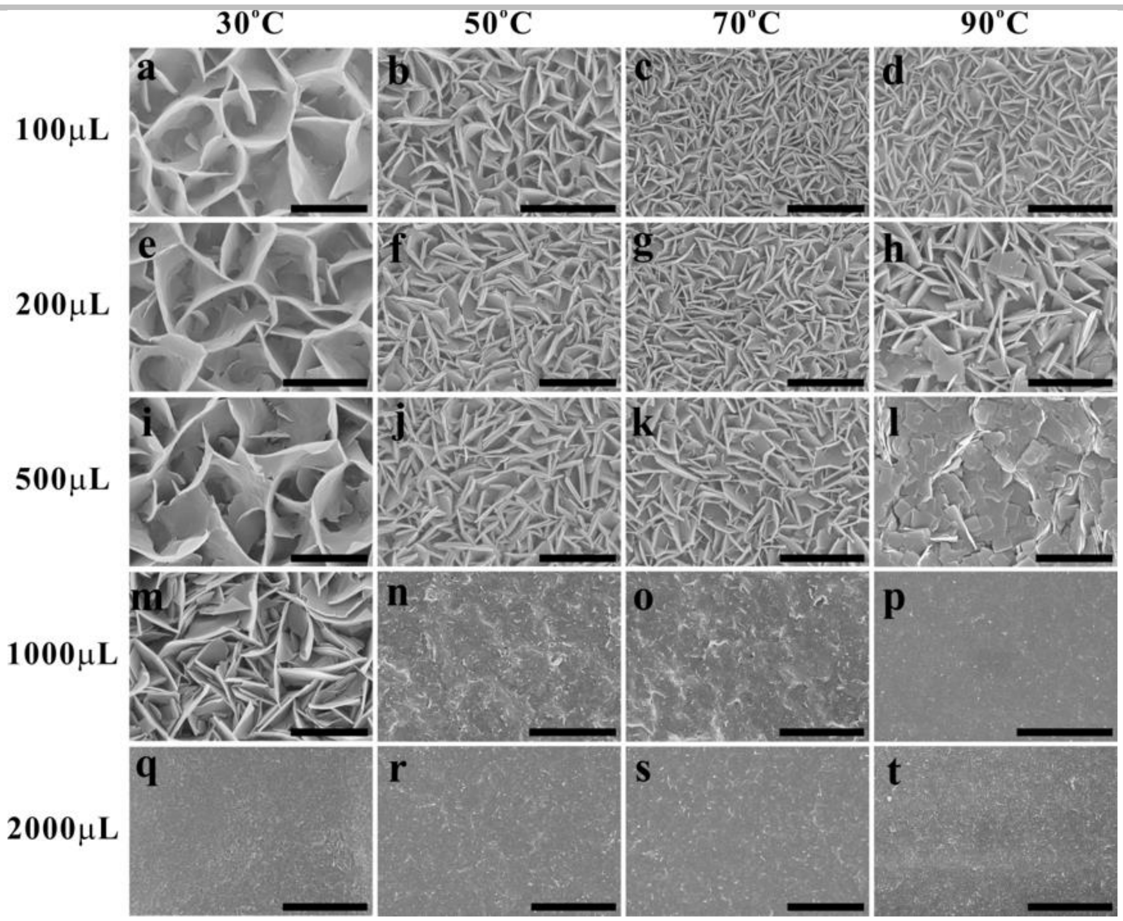

Figure S8. (a-t) Surface SEM images of the CuBDC products synthesized with different solvent composition (vertical ordinate, the amount of water in 10 mL DMF) and reaction temperature (horizontal ordinate). All of the concentrations of organic ligands for a-t are 0.2 g L^{-1}. Scare bars represent 2 μm for (a-t).

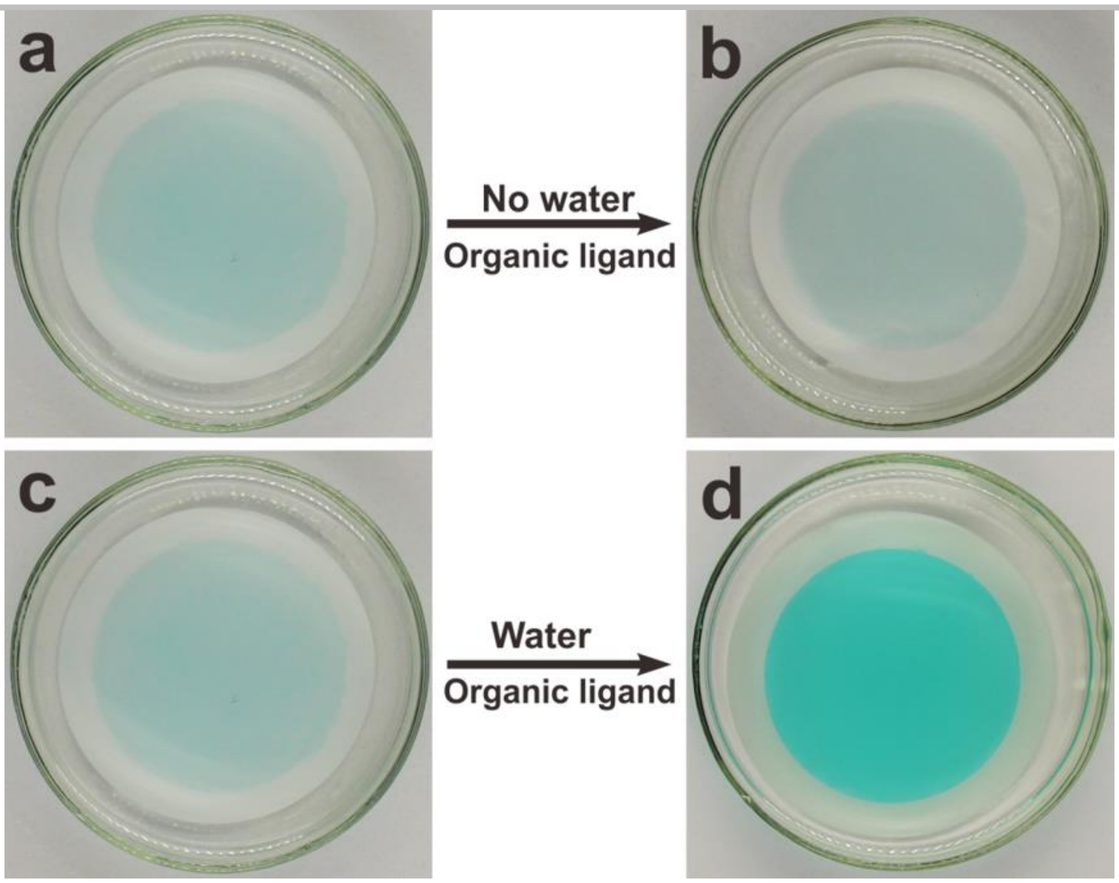

Figure S9. (a-d) Photographs of products synthesized with water and without water, respectively.

The etching ability of terephthalic acid in DMF is too poor to release enough concentration of Cu^{2+} for the formation of CuBDC. To increase the dissolution rate of $Cu(OH)_2$, water molecules are designedly introduced as solvent in the synthetic process. Consequently, the coordination process takes place in the mixed solvent proved the water molecules playing important role in the formation of CuBDC.

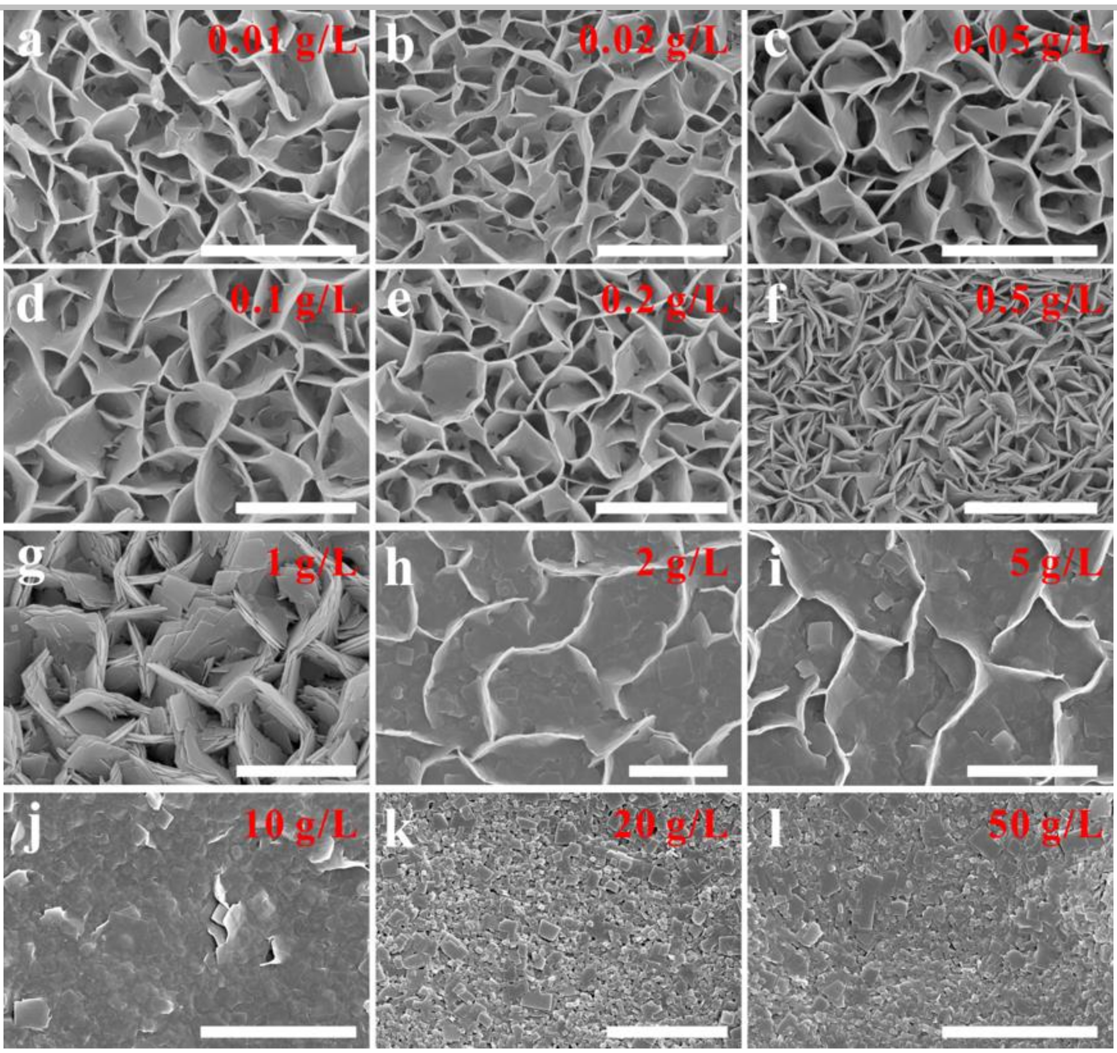

Figure S10. (a-l) Surface SEM images of CuBDC film which were constructed at different concentration organic ligands (from 0.01 g L^{-1} to 50 g L^{-1}). Scare bars represent 3 μm for (a-l). The reaction conditions are water/DMF (v:v = 0.01) and 30 ^{o}C.

The SEM images demonstrated that the CuBDC NAF structure tend to emerged at low concentration of organic ligands (0.01 g L^{-1} - 0.2 g L^{-1}) while the CuBDC NAF structure formed at high concentration of organic ligands (10 g L^{-1} - 50 g L^{-1}).

Supplementary Note 1. The reactions for the construction of CuBDC crystals:

The conversion of $Cu(OH)_2$ into CuBDC bulk:

$$\mathbf{Cu(OH)_2 \leftrightharpoons Cu^{2+} + 2OH^-} \quad (1)$$

$$\mathbf{Cu^{2+} + H_2BDC \leftrightharpoons Cu(H_2BDC)^{2+}} \quad (2)$$

$$\mathbf{Cu(H_2BDC)^{2+} + 2OH^- \leftrightharpoons Cu(BDC) + 2H_2O} \quad (3)$$

The total reaction as follow: $\mathbf{Cu(OH)_2 + H_2BDC \leftrightharpoons Cu(BDC) + 2H_2O}$

The conversion of $Cu(OH)_2$ into CuBDC NAF:

$$\mathbf{Cu(OH)_2 + H_2BDC \leftrightharpoons Cu(BDC) + 2H_2O} \quad (4)$$

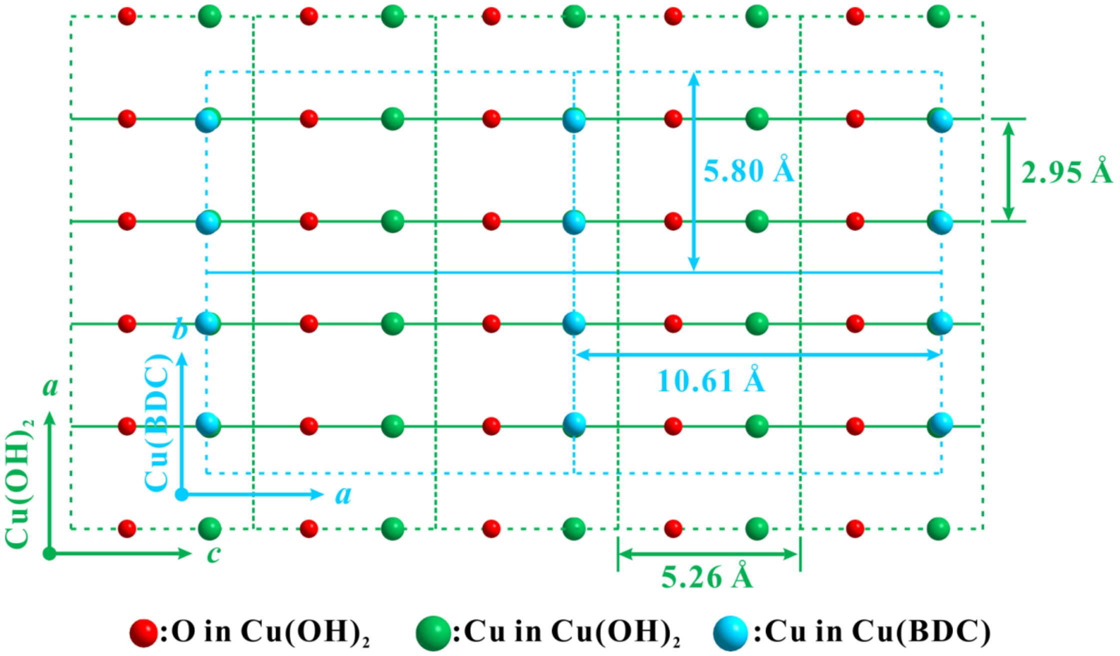

Figure S11. Schematic illustration showing crystal lattice of $Cu(OH)_2$ and Cu(BDC) from ***ac*** and ***ab*** projections, respectively. Cu positions are highlighted by different colors for both crystals (green for $Cu(OH)_2$ and cyan for Cu(BDC). These values indicate that $Cu(OH)_2$ and Cu(BDC) MOFs provide lattice matching conditions for heteroepitaxial growth.

Copper hydroxide possesses lattice parameters:[6]

Cmc2₁ space group, $\boldsymbol{a}$ = 2.95 Å, $\boldsymbol{b}$ = 10.59 Å and $\boldsymbol{c}$ = 5.26 Å.

CuBDC possesses lattice parameters:

P4 space group, $\boldsymbol{a}$ = 10.61 Å, $\boldsymbol{b}$ = 5.80 Å and $\boldsymbol{c}$ = 10.61 Å.

During the heteroepitaxial growth process, the ***a*** and ***b*** axis of MOF crystals would align with the ***c*** and ***a*** axis of a $Cu(OH)_2$ substrate, respectively. Finally the $Cu(OH)_2$ substrate was readily convert into CuBDC nanosheets via orientated growth.

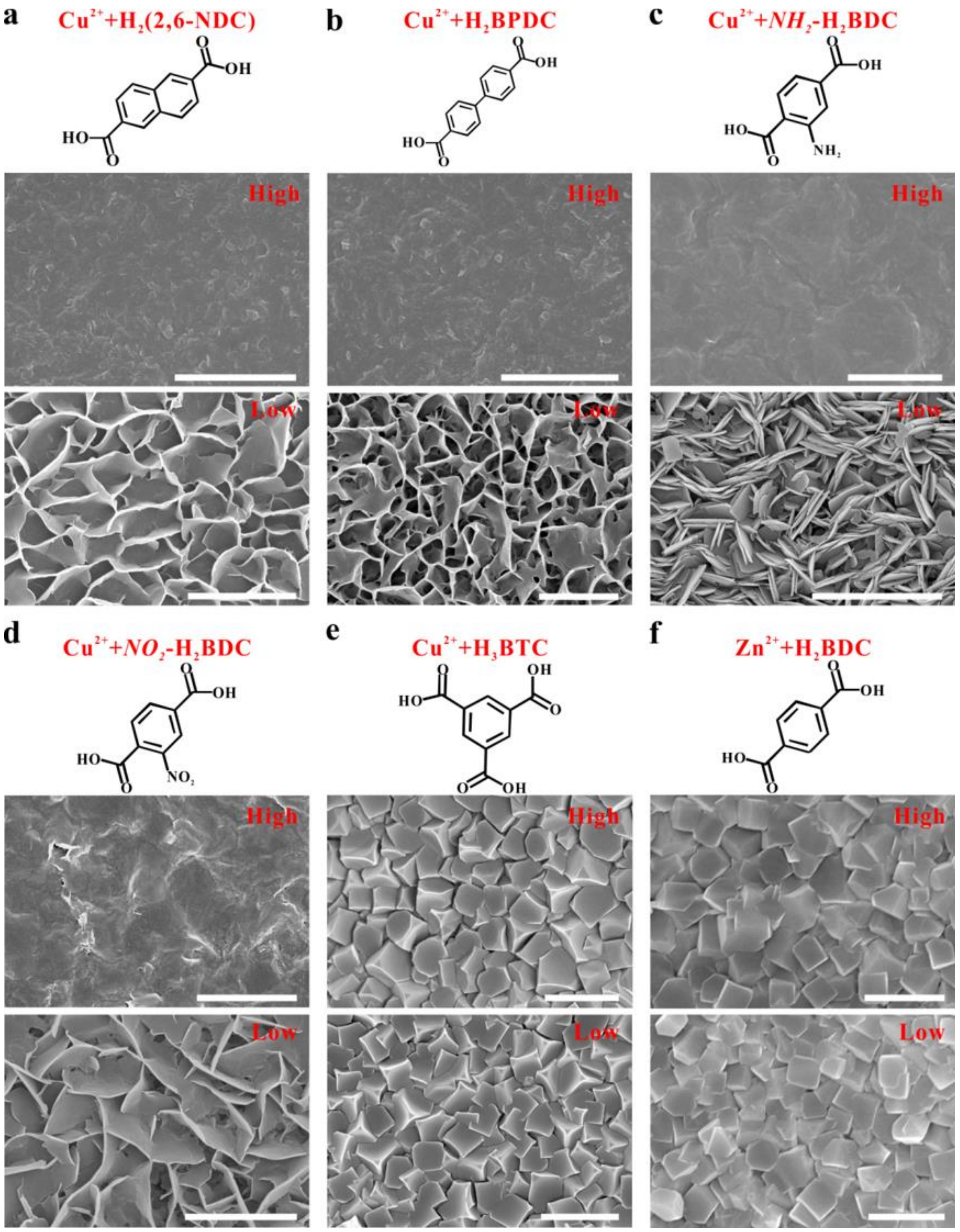

Figure S12. (a-e) The metal cations, the organic ligands and the surface SEM images of the MOFs prepared at high (top) and low (bottom) concentration of organic ligands. The high concentrations and low concentrations are 5 g L^{-1} and 0.2 g L^{-1} for (a) to (f), respectively. Scare bars represent 3 μm for (a-f).

Only when the lattice mismatch between the the MOF crystal and the inorganic substrate is less than 1.8%, the heteroepitaxial growth process occured. The values of lattice mismatch were calculated between the ***a*** axis of MOFs and the ***c*** axis of $Cu(OH)_2$.

For example, the lattice mismatch between Cu(2,6-NDC), Cu(BPDC) and copper hydroxide are 1.5% and 1.8, respectively, so the Cu(2,6-NDC) and Cu(BPDC) NAF films can be obtained via orientated growth. The lattice mismatch between HKUST-1 (*Fm-3m* space group, ***a*** = 26.34 Å, ***b*** = 26.34 Å and ***c*** = 26.34 Å),[7] MOF-5 (*Fm-3m* space group, ***a*** = 25.8 Å, ***b*** = 25.8 Å and ***c*** = 25.8 Å)[8] and their coorsponding ceramic materials (Copper hydroxide: *Cmc2₁* space group, ***a*** = 2.95 Å, ***b*** = 10.59 Å and ***c*** = 5.26 Å. Zinc hydroxide: *P-3m1* space group, ***a*** = 3.19 Å, ***b*** = 3.19 Å and ***c*** = 4.65 Å)[9] are too large, so no orientated growth occured.

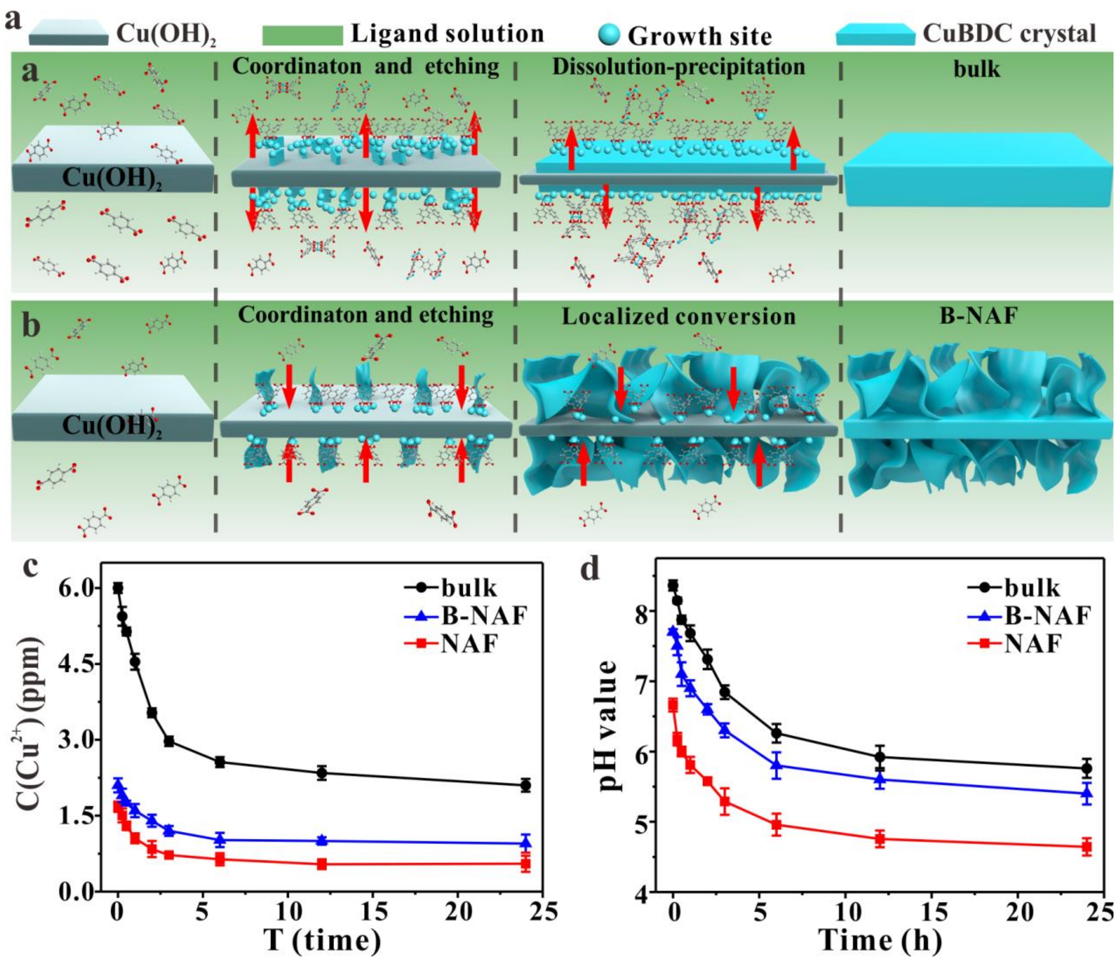

Figure S13. (a) Schematic view of the mechanism for the preparation of CuBDC B-NAF; (b) Schematic view of the mechanism for the preparation of CuBDC bulk; (c, d) Concentration of Cu^{2+} (left) and pH value (right) versus reaction time of the reaction at different concentrations of organic ligands, respectively. The reaction conditions are water/DMF (v:v = 0.01) and 30 °C. Error bars represent the standard deviation of three replicate samples.

Supplementary Note 2. The mechanisms for the preparation of CuBDC crystals:

For the low concentration of ligands, as dissolution and coordination rate are too low for the concentration of $Cu(H_2BDC)^{2+}$ in the solvent to reach the nucleation concentration of CuBDC, the nucleation of CuBDC crystal is hindered. The formation of CuBDC crystal is prone to take place on the surface of substrate by a direct localized conversion without any release of copper cations into the solution. For the high concentration of ligands, a large amount of copper cations were released from the surface of copper hydroxide film and coordinated by H_2BDC to form $Cu(H_2BDC)^{2+}$. The CuBDC nucleated from the oversaturated solution of $Cu(H_2BDC)^{2+}$ and coated onto the surface of templates. Finally the CuBDC crystals grew with the reaction proceeds and connected to each other to form an integrated bulk film.

The initial $Cu(H_2BDC)^{2+}$ concentration was 6.02 ppm when the $Cu(OH)_2$ templates were added into the high ligand concentration. This concentration quickly decreased within 2 h, indicating the rapid consumption of $Cu(H_2BDC)^{2+}$ in the early stages. The decrease in the $Cu(H_2BDC)^{2+}$ concentration slowed at about 2 h, and remained at about 2.56 ppm after 6 h, indicating that the reactions had reached equilibrium. The initial $Cu(H_2BDC)^{2+}$ concentration in solvent for NAF (1.68 ppm) is much lower than the initial ligand concentration (6.02 ppm), and even lower than the equilibrium concentration (2.10 ppm) after 24 h for the high ligand concentration. The $Cu(H_2BDC)^{2+}$ concentration in the solution decreased dramatically within 3 h (0.72 ppm). This process was considered as nucleation of CuBDC crystals from the solution, which were deposited onto the surface of the templates. Afterwards, the $Cu(H_2BDC)^{2+}$ concentration remained low (~0.5 ppm), indicating that the whole process takes place without any release of metal ions into the solution. As shown in Fig. 4j, the pH values were also tracked during the reaction, and were found to gradually decrease as the concentration of $Cu(H_2BDC)^{2+}$ decreased, which can be attributed to an acid–base interaction between the $Cu(OH)_2$ and H_2BDC.

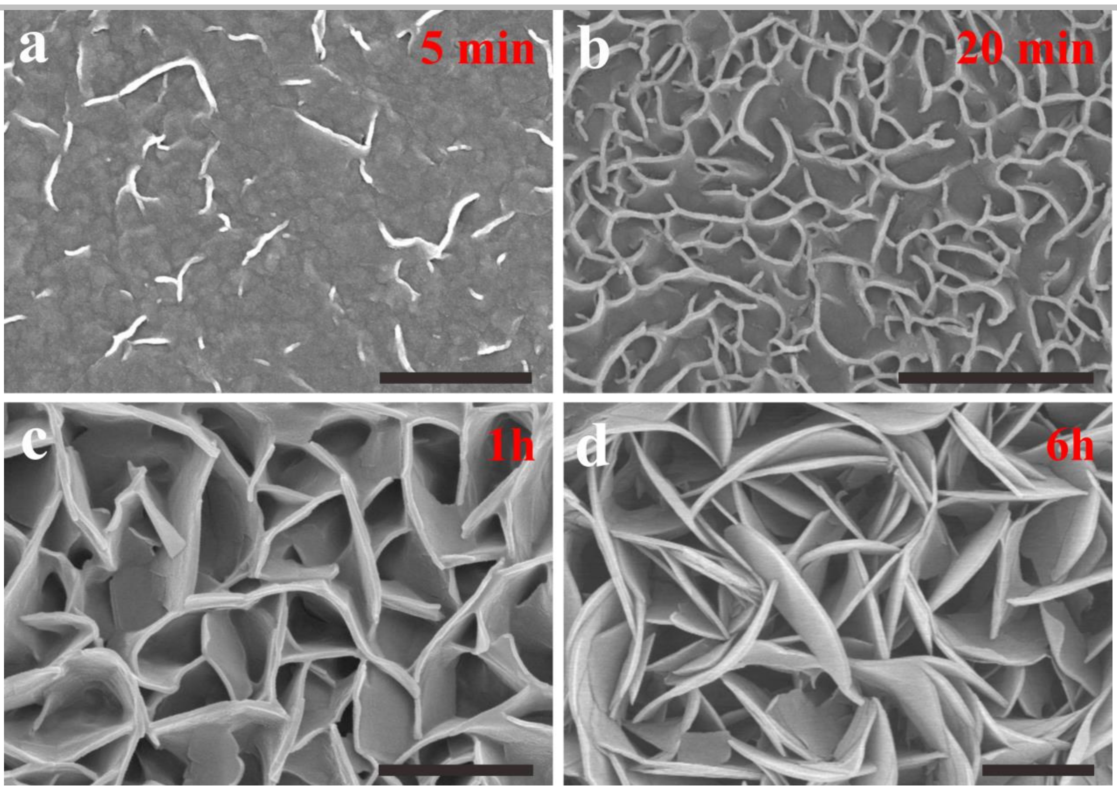

Figure S14. (a-d) Surface SEM images of copper hydroxide nanostrands thin film was transformed into CuBDC B-NAF at different reaction time; Scale bars were 1 μm for a-d.

The nanosheets have covered the surface of the film after 20 min of reaction at room temperature. The copper hydroxide nanostrands thin film has transformed into CuBDC B-NAF completely at 3 h. Compared with the growth of NAF, it's obvious that the conversion rate for CuBDC B-NAF is much higher, and the nanosheets are much more close-packing to cover the surface of thin film.

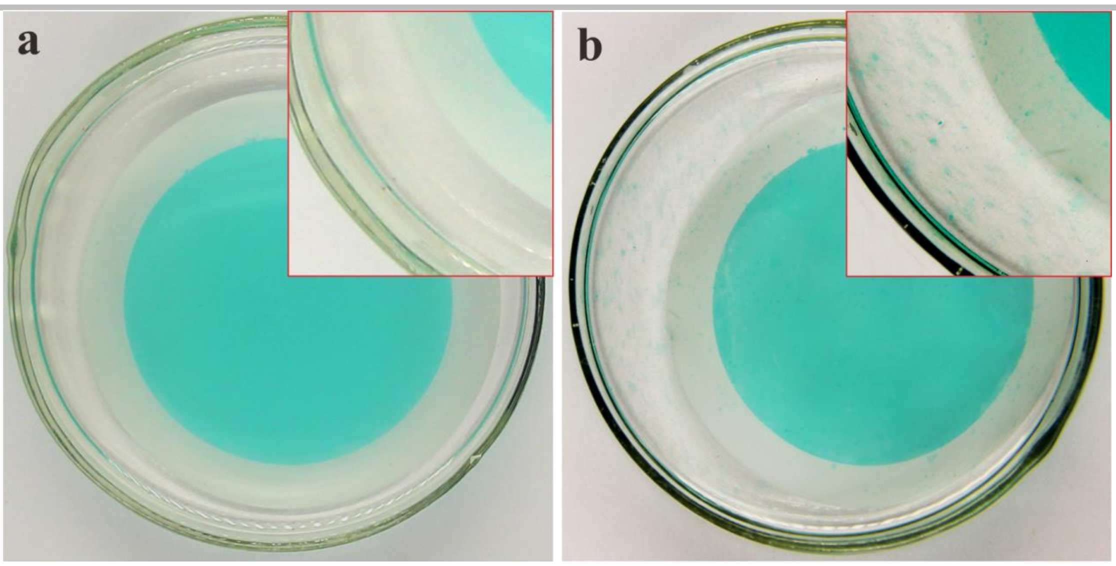

Figure S15. (a, b) Photographs of as-synthesized CuBDC coated nylon 66 membrane synthesized with low and high concentration of organic ligand, respectively.

It's obvious that there are a lot of blue CuBDC crystals in the solvent in high concentration of organic ligand while not in low concentration of organic ligand. The conversion of $Cu(OH)_2$ into CuBDC bulk crystals is followed by the 'dissolution-precipitation mechanism' at high ligand concentration, resulting in CuBDC crystals that are nucleated in the solvent. The conversion of $Cu(OH)_2$ into CuBDC NAF crystals is followed by the 'heteroepitaxial growth mechanism' at low ligand concentration; thus, there is no CuBDC crystals was observed in the solvent.

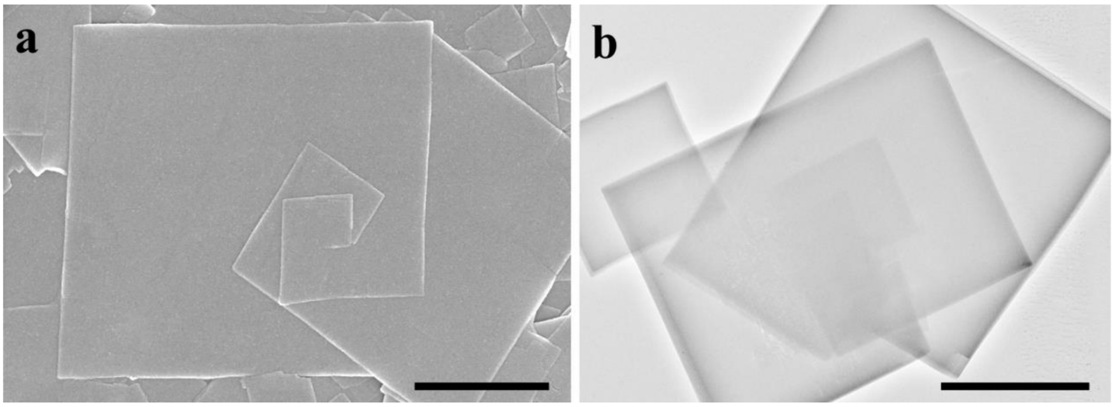

Figure S16. (a) The surface SEM image of the NSs for the test film samples on Si/SiO_2 substrates. (b) The TEM for the NSs. Scale bars represent 1 μm for (a) and (b).

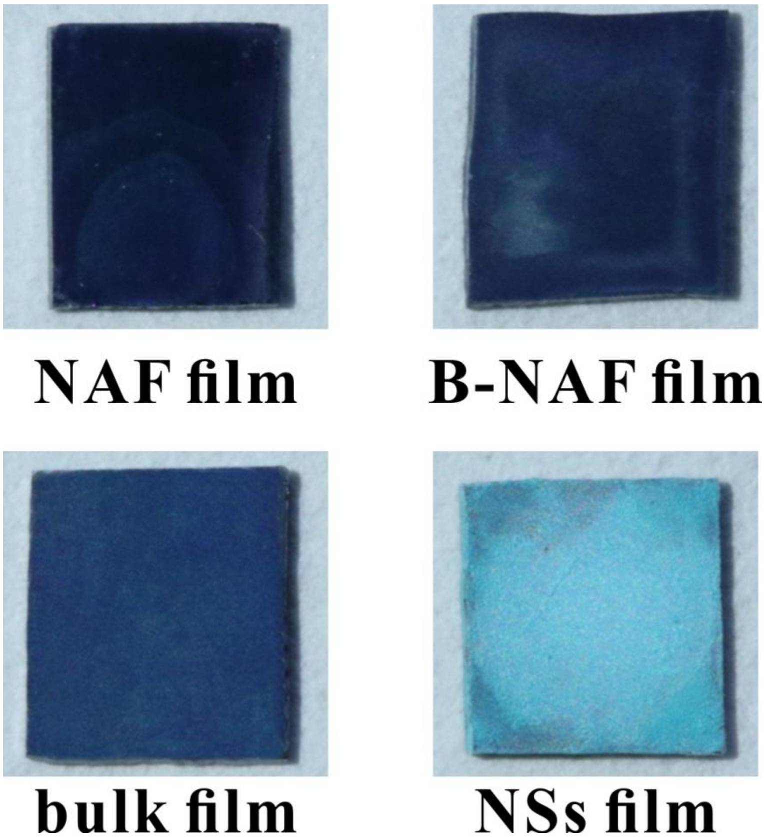

Figure S17. Photographs of the test film samples on Si/SiO_2 substrates. The size of the silicon wafer is ~1.0 cm×1.0 cm.

Optical images of the four CuBDC films on silicon wafers present different colors. Based on the refractive index and thickness, the reflected light peak position can be acquired using the following equation:

$$\lambda = 2nh/m$$

where λ, n, h, and m are the peak position, refractive index, thickness, and an integer number representing the peak index, respectively. It is obvious the thickness for the four CuBDC films are different.

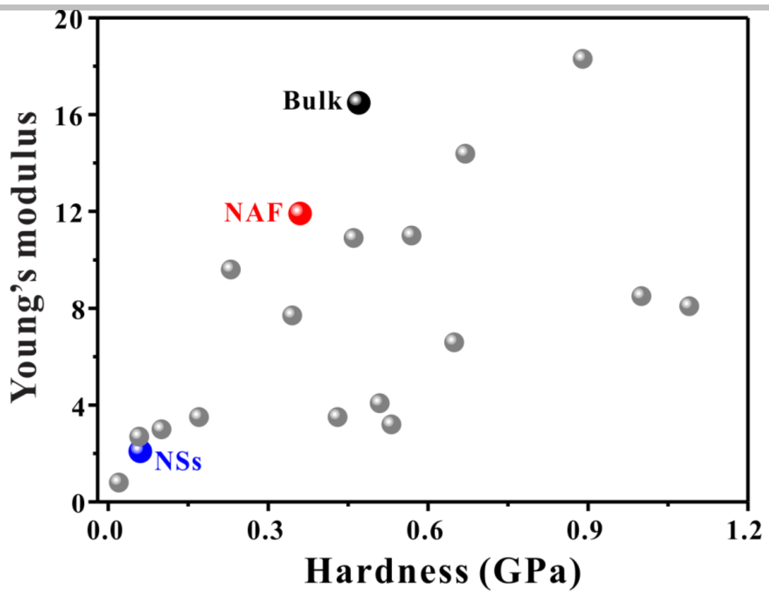

Figure S18. Hardness and Young's modulus of NAF in comparison with various MOFs materials (details see Table 2 in the Supporting Information).

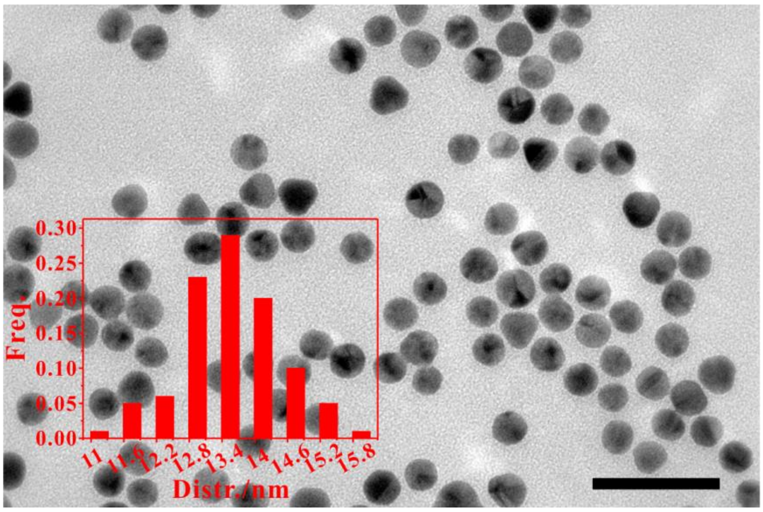

Figure S19. TEM image of Au NPs, the inset image shows a size distribution for as-synthesized Au NPs. Scare bar is 50 nm.

Transmission electron microscopy (TEM) study suggests that the Au NPs have a well-defined structure with sizes of 13 ± 1 nm.

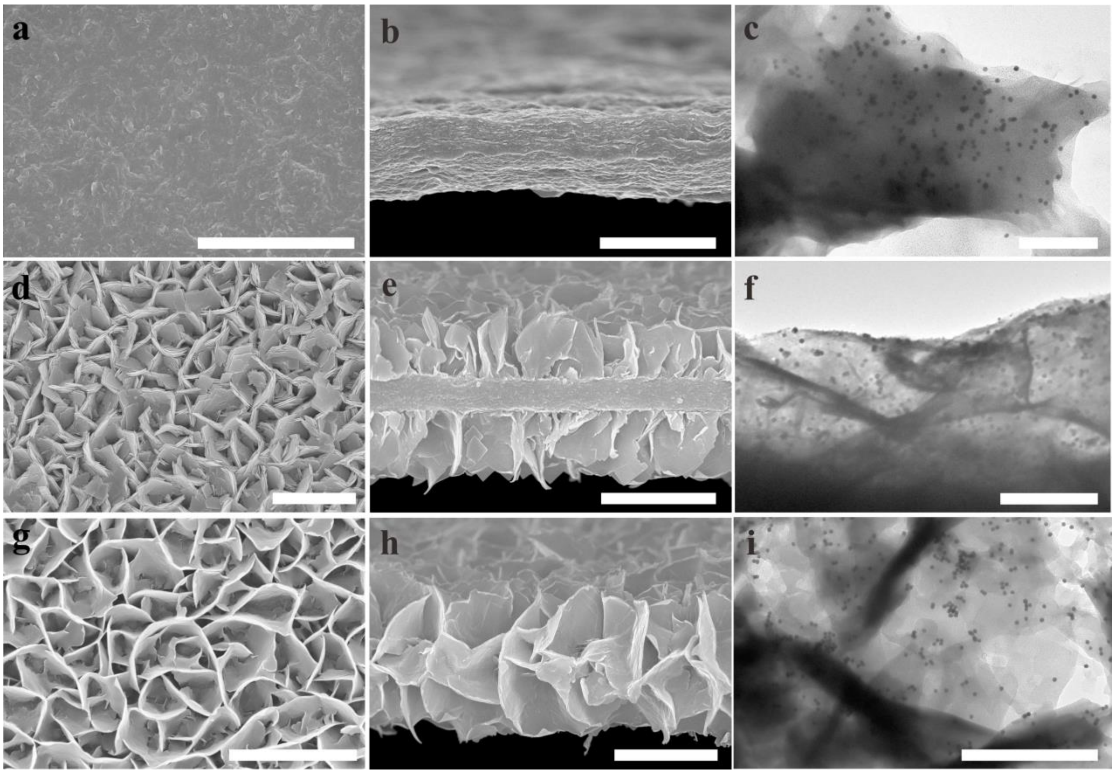

Figure S20. (a, b, c) Surface and cross-section SEM and TEM images of Au NPs@ bulk; (d, e, f) Surface and cross-section SEM and TEM images of Au NPs@B-NAF; (g, h, i) Surface and cross-section SEM and TEM images of Au NPs@ NAF. Scare bars represent 5 µm for a, d, g; 2 µm for b, e, h; 500 nm for c, f, i.

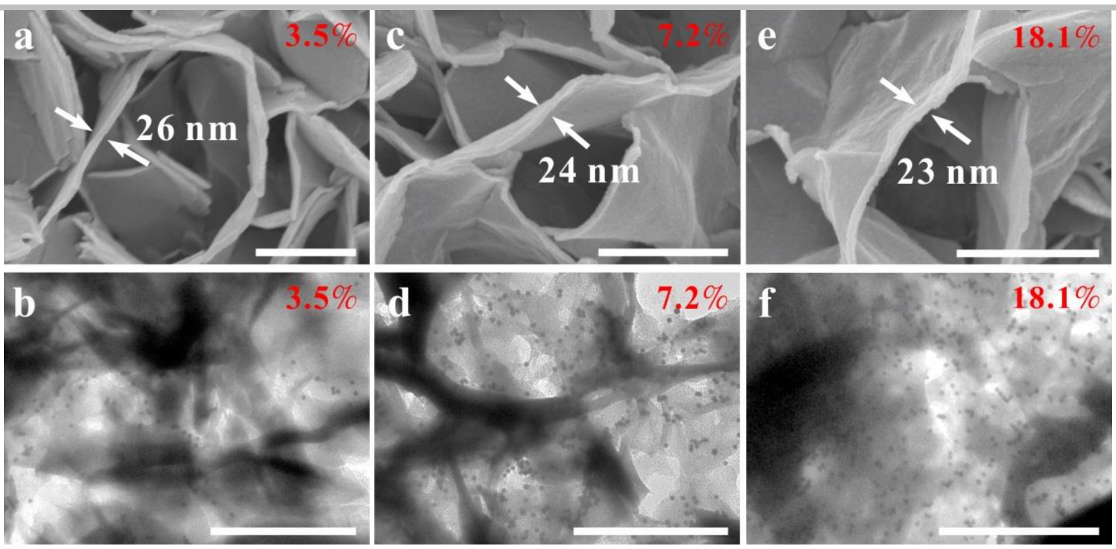

Figure S21. (a-g) Surface SEM and TEM images for the Au NPs@NAF with the loaded amount of 3.5% Au NPs; (i-j) Surface SEM and TEM images for the Au NPs @NAF with the loaded amount of 7.2% Au NPs; (g-h) Surface SEM and TEM images for the Au NPs @NAF with the loaded amount of 18.1% Au NPs. Scare bars represent 500 nm for a-f.

The surface of Au NPs@NAF nanosheets is smooth and the thickness of all the Au NPs@NAF nanosheets is ~25 nm which is not affected by the loaded amount of Au NPs. Well-dispersed Au NPs were fully encapsulated within the CuBDC nanosheets, and no Au NPs were observed on the outside surface of the CuBDC. The above results exhibit that this strategy could be used to construct Au NPs@NAF composites.

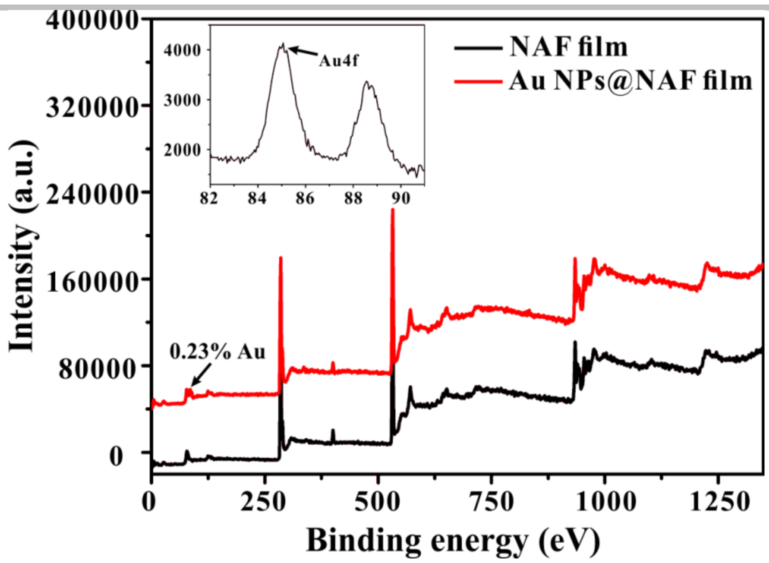

Figure S22. XPS spectra of the surface of as-synthesized CuBDC NAF and Au NPs@CuBDC NAF films.

There is only 0.23% of Au atoms are detected, suggesting almost all of the Au NPs are encapsulated into CuBDC NSs. It is well known that the effective detection depth of XPS is ~5 nm, the thickness of the NSs in Au NPs@NAF films is only ~25 nm. So it is reasonable to think that almost all of the Au NPs are well encapsulated into CuBDC NSs and only very few of Au atoms could be detected by XPS.

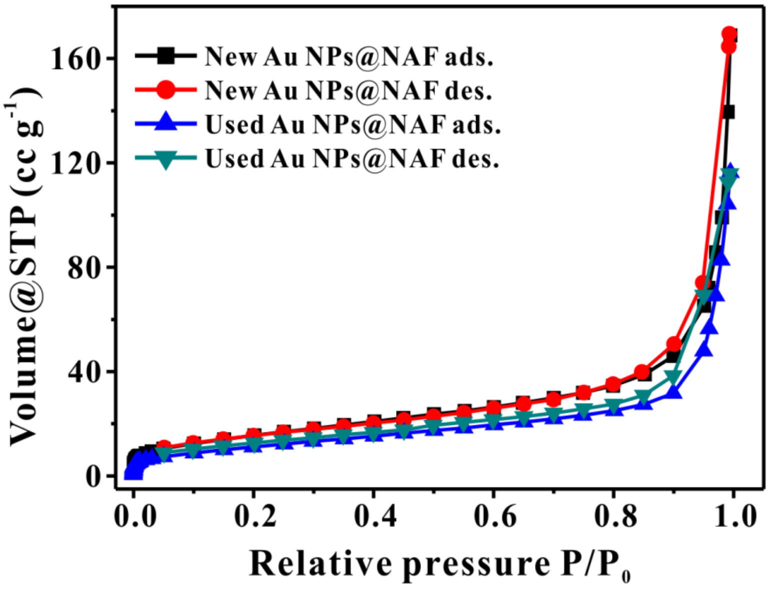

Figure S23. Nitrogen adsorption and desorption isotherms measured at 77 K of new-synthesized CuBDC NAF and used CuBDC NAF.

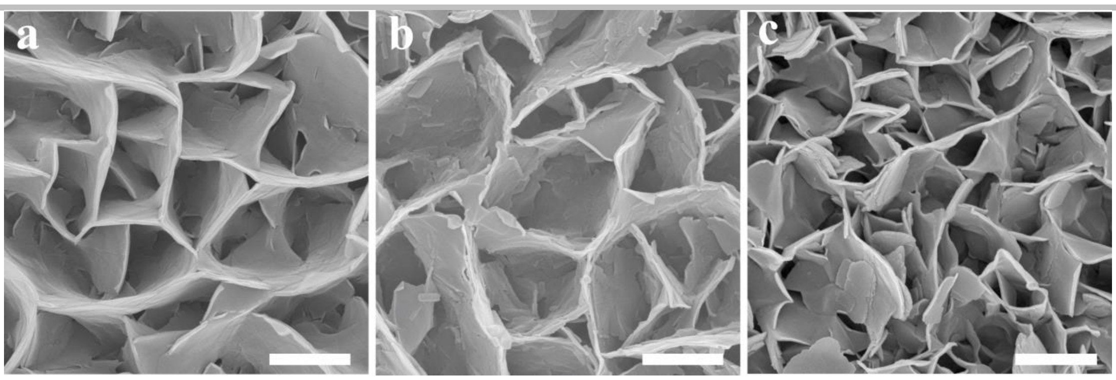

Figure S24. (a) Surface SEM image for as-synthesized Au NPs@ NAF; (b-c) Surface SEM images for Au NPs@NAF after reaction at 190 ˚C for up to 10 h and 30 h. Scare bars represent 1 μm for (a-c).

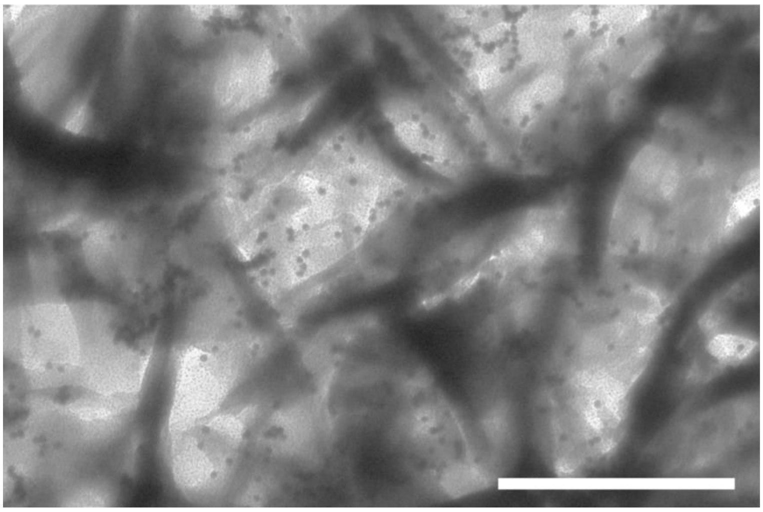

Figure S25. TEM images for Au NPs@ NAF after reaction at 190 ˚C for up to 30 h. Scare bar represents 500 nm.

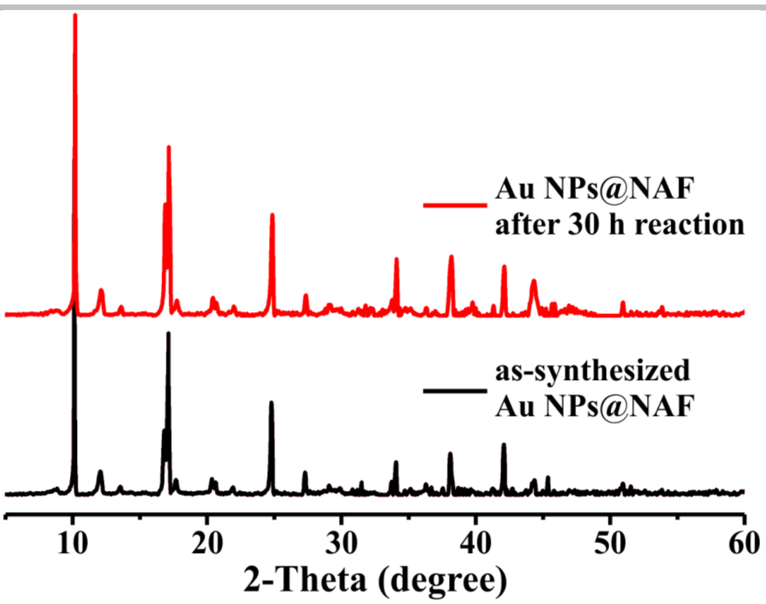

Figure S26. Powder XRD patterns for as-synthesized Au NPs@CuBDC NAF and experimental Au NPs@NAF after 30 h CO oxidation reaction.

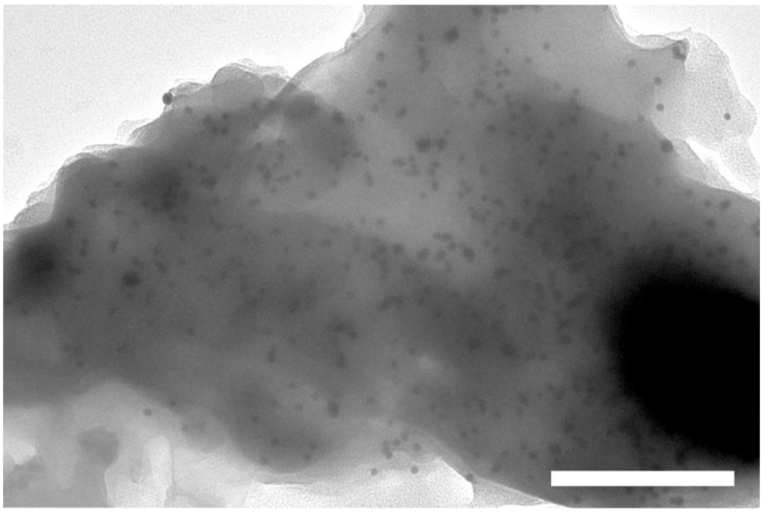

Figure S27. TEM images for Au NPs@bulk after reaction at 190 ˚C for up to 30 h. Scare bar represents 300 nm.

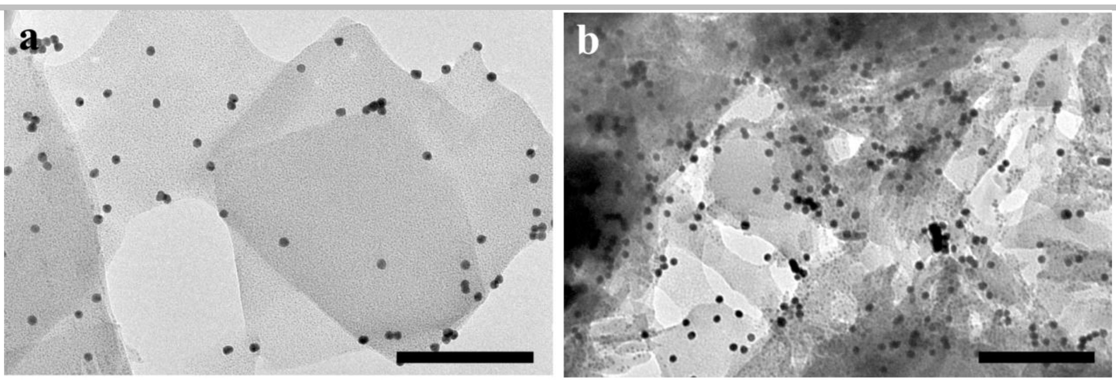

Figure S28. (a, b) TEM images for Au NPs/CuBDC NSs before and after reaction at 190 ˚C for up to 30 h. Scare bars represent 200 nm for (a) and (b).

Table S1. Complete results of the nanoindentation test for different CuBDC at various load.

**Materials**	**Load (uN)**	**Young's modulus (GPa)**	**Hardness (GPa)**	**Indentation depth (nm)**
CuBDC bulk film	500	13.56	0.23	263.37
		14.54	0.44	195.06
		15.13	0.42	197.85
		15.77	0.57	173.50
		17.01	0.47	185.57
		17.63	0.49	181.70
		21.74	0.70	151.67
Average value		16.48	0.47	192.67
CuBDC NAF film	500	10.75	0.35	223.16
		10.87	0.28	246.32
		10.96	0.32	231.84
		11.57	0.34	224.64
		11.98	0.30	234.49
		12.72	0.39	208.30
		14.51	0.51	182.97
Average value		11.91	0.36	221.68
CuBDC NS film	200	1.68	0.04	411.93
		1.70	0.05	398.42
		1.71	0.03	483.74
		2.13	0.07	319.06
		2.39	0.09	285.22
		2.49	0.04	410.63
		2.49	0.07	324.83
Average value		2.09	0.06	376.26

Table S2. Comparison of mechanical properties for various reported MOFs materials.

	Name	Young's modulus (GPa)	Hardness (GPa)	Reference
1	CuBDC bulk film	16.48	0.47	**This work**
2	CuBDC NAF film	11.91	0.36	
3	CuBDC NS film	2.09	0.06	
4	MOF-5	2.7	0.058	[10]
5	*a*-ZIF	6.59	0.649	[11]
6	ZIF-4	4.08	0.509	
7	ZIF-zni	8.09	1.09	
8	ZIF-8	3.199	0.531	[12]
9	HKUST-1 film	9.6	0.23	[13]
10	Mg/DOBDC	11	0.569	[14]
11	Ni/DOBDC	7.7	0.345	
12	ZIF-zni $Zn(Im)_2$	8.5	1	[15]
13	BIF-1-Li	3	0.1	
14	ZIF-8 films	3.5	0.43	[16]
15	Cu(CHDA)	10.9	0.46	[17]
16	$Cu(INA)_2$	0.8	0.02	
17	$Cu_3(BTC)_2$	3.5	0.17	
18	$[Cu_2F(HF)(HF_2)(pyz)_4][(SbF_6)_2]_n$	14.39	0.67	[18]
19	Mn(2-methylsuccinate)	18.3	0.89	[19]

References

[1] Y. Mao, J. Li, W. Cao, Y. Ying, P. Hu, Y. Liu, L. Sun, H. Wang, C. Jin, X. Peng, *Nat. Commun.* **2014**, 5, 5532.
[2] G. Xu, K. Otsubo, T. Yamada, S. Sakaida, H. Kitagawa, *J. Am. Chem. Soc.* **2013**, 135, 7438.
[3] G. Xu, T. Yamada, K. Otsubo, S. Sakaida, H. Kitagawa, *J. Am. Chem. Soc.* **2012**, 134, 16524.
[4] R. Sakamoto, K. Hoshiko, Q. Liu, T. Yagi, T. Nagayama, S. Kusaka, M. Tsuchiya, Y. Kitagawa, W.-Y. Wong, H. Nishihara, *Nat. Commun.* **2015**, 6, 6713.
[5] Y. Wang, M. Zhao, J. Ping, B. Chen, X. Cao, Y. Huang, C. Tan, Q. Ma, S. Wu, Y. Yu, Q. Lu, J. Chen, W. Zhao, Y. Ying, H. Zhang, *Adv. Mater.* **2016**, 28, 4149.
[6] H.-R. Oswald, A. Reller, H. Schmalle, E. Dubler, *Acta Cryst.* **1990**, 46, 2279.
[7] S. S.-Y. Chui, S. M.-F. Lo, J. P. Charmant, A. G. Orpen, I. D. Williams, *Science* **1999**, 283, 1148.
[8] J. L. Rowsell, E. C. Spencer, J. Eckert, J. A. Howard, O. M. Yaghi, *Science* **2005**, 309, 1350.
[9] S. Cherepanova, D. Markovskaya, E. Kozlova, *Acta Crystallogr., Sect. B: Struct. Sci.* **2017**, 73, 360.
[10] D. F. Bahr, J. A. Reid, W. M. Mook, C. A. Bauer, R. Stumpf, A. J. Skulan, N. R. Moody, B. A. Simmons, M. M. Shindel, M. D. Allendorf, *Phys. Rev. B: Condens. Matter Mater. Phys.* **2007**, 76, 184106.
[11] T. D. Bennett, A. L. Goodwin, M. T. Dove, D. A. Keen, M. G. Tucker, E. R. Barney, A. K. Soper, E. G. Bithell, J.-C. Tan, A. K. Cheetham, *Phys. Rev. Lett.* **2010**, 104, 115503.
[12] J. C. Tan, T. D. Bennett, A. K. Cheetham, *Proc. Natl. Acad. Sci. USA* **2010**, 107, 9938.
[13] S. Bundschuh, O. Kraft, H. K. Arslan, H. Gliemann, P. G. Weidler, C. Wöll, *Appl. Phys. Lett.* **2012**, 101, 101910.
[14] R. Kumar, D. Raut, U. Ramamurty, C. N. R. Rao, *Angew. Chem., Int. Ed.*, **2016**, 55, 7857.
[15] T. D. Bennett, J.-C. Tan, S. A. Moggach, R. Galvelis, C. Mellot-Draznieks, B. A. Reisner, A. Thirumurugan, D. R. Allan, A. K. Cheetham, *Chem.–Eur. J.*, **2010,** 16, 10684.
[16] S. Eslava, L. Zhang, S. Esconjauregui, J. Yang, K. Vanstreels, M. R. Baklanov, E. Saiz, *Chem. Mater.* **2013**, 25, 27.
[17] B. Van de Voorde, R. Ameloot, I. Stassen, M. Everaert, D. De Vos, J.-C. Tan, *J. Mater. Chem.* C **2013**, 1, 7716.
[18] W. Li, M. S. R. N. Kiran, J. L. Manson, J. A. Schlueter, A. Thirumurugan, U. Ramamurty, A. K. Cheetham, *Chem. Commun.* **2013**, 49, 4471.
[19] W. Li, P. T. Barton, M. S. R. N. Kiran, R. P. Burwood, U. Ramamurty, A. K. Cheetham, *Chem.–Eur. J.*, **2011**, 17, 12429
